# The two faces of cyanide: an environmental toxin and a potential novel mammalian gasotransmitter

**DOI:** 10.1111/febs.16135

**Published:** 2021-08-05

**Authors:** Karim Zuhra, Csaba Szabo

**Affiliations:** ^1^ Chair of Pharmacology Section of Medicine University of Fribourg Switzerland

**Keywords:** bioenergetics, carbon monoxide, hydrogen sulfide, metabolism, mitochondria, nitric oxide

## Abstract

Cyanide is traditionally viewed as a cytotoxic agent, with its primary mode of action being the inhibition of mitochondrial Complex IV (cytochrome c oxidase). However, recent studies demonstrate that the effect of cyanide on Complex IV in various mammalian cells is biphasic: in lower concentrations (nanomolar to low micromolar) cyanide stimulates Complex IV activity, increases ATP production and accelerates cell proliferation, while at higher concentrations (high micromolar to low millimolar) it produces the previously known (‘classic’) toxic effects. The first part of the article describes the cytotoxic actions of cyanide in the context of environmental toxicology, and highlights pathophysiological conditions (e.g., cystic fibrosis with *Pseudomonas* colonization) where bacterially produced cyanide exerts deleterious effects to the host. The second part of the article summarizes the mammalian sources of cyanide production and overviews the emerging concept that mammalian cells may produce cyanide, in low concentrations, to serve biological regulatory roles. Cyanide fulfills many of the general criteria as a ‘classical’ mammalian gasotransmitter and shares some common features with the current members of this class: nitric oxide, carbon monoxide, and hydrogen sulfide.

## Graphical abstract

Role of low‐ and high‐cyanide concentration in mammalian cell function regulation in health and disease. At low (nanomolar to low micromolar) concentrations, cyanide exerts beneficial and regulatory roles. At medium concentrations, the effect of cyanide includes the regulation of NMDA receptors and the modulation of intracellular calcium handling. At high (high micromolar to millimolar) concentrations, cyanide induces calcium overload and mitochondrial dysfunction, culminating in apoptotic or necrotic cell death.

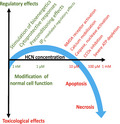

**Abbreviations**
3‐MST: 3‐mercaptopyruvate sulfurtransferase
ACC: 1‐aminocyclopropane‐1‐carboxylic acid
AdoCbl: 5’‐deoxyadenosylcobalamin
ATCA: 2‐aminothiazoline‐4‐carboxylic acid
BAX: BCL‐2‐associated X protein
BNIP3: BCL2 and adenovirus E1B 19‐kDa‐interacting protein 3
CBS: cystathionine‐β‐synthase
CCOx: cytochrome c oxidase
CF: cystic fibrosis
CNCbl: cyanocobalamin
CNS: central nervous system
CO: carbon monoxide
CO_2_
: carbon dioxide
CSE: cystathionine‐γ‐lyase
eNOS: endothelial nitric oxide synthase
H_2_O_2_
: hydrogen peroxide
H_2_S: hydrogen sulfide
HIF‐1α: hypoxia‐inducible factor‐1α
IP_3_
: inositol triphosphate
KCN: potassium cyanide
MeCbl: methylcobalamin
MPO: myeloperoxidase
NMDA: N‐methyl‐d‐aspartic acid
NO: nitric oxide
O_2_
: oxygen
PARP: poly(ADP‐ribose) polymerase
PKC: protein kinase C
ROS: reactive oxygen species
SCN: thiocyanate
SOD: superoxide dismutase
SQR: sulfide:quinone oxidoreductase
TST: thiosulfate sulfurtransferase (rhodanese)
VEGF: vascular endothelial growth factor

## Introduction

Cyanide is endogenously produced in organisms from many kingdoms including bacteria, fungi, arthropods, and plants. In these organisms, cyanide serves various important biological regulatory functions. For instance, in cyanide‐producing bacteria (e.g., *Pseudomonas aeruginosa*), cyanide is known to play a role in quorum sensing and may act as a virulence factor and a mediator of biotic interactions [1, 2, 3, 4, 5].

In various plants, endogenously produced cyanide has been implicated in seed germination, plant development and in plant immunity [6, 7, 8, 9, 10].

In bacteria, cyanide is produced from glycine in an oxidative reaction catalyzed by the enzyme cyanide synthase, also known as glycine dehydrogenase (cyanide‐forming). In plants, cyanide production is part of the camalexin or ethylene biosynthetic pathways. In one reaction, the cysteine‐indole‐3‐acetonitrile conjugate acts as the substrate for CYP71B15(PAD3) to produce hydrogen cyanide and dihydrocamalexic acid (in a subsequent step, the latter product is converted to camalexin). In another reaction that occurs in many plant species, 1‐aminocyclopropane‐1‐carboxylic acid (ACC) acts as the substrate of ACC oxidase, with the products being ethylene, carbon dioxide, and hydrogen cyanide.

In contrast to bacteria and plants, for mammalian cells, cyanide is usually viewed as an environmental toxin and a poison. (In fact, the Swedish chemist Carl Wilhelm Scheele, who first isolated hydrogen cyanide, has reportedly died from the adverse health effects of cyanide poisoning in 1786). The environmental toxicology of cyanide has an extensive literature, focusing on industrial, environmental and alimentary sources, exposure levels, absorption, organ distribution, elimination and on the various therapeutic options (antidotes). These topics are the subject of specialized reviews [11, 12, 13, 14, 15]. Environmental sources of cyanide include various industrial processes, smoking and fires, intentional poisoning, and certain natural, alimentary sources of cyanides (over 2000 plant species, including cassava and many fruits and vegetables containing cyanogenic glycosides, almonds, and apricot kernels). There is also a significant iatrogenic source of cyanide: sodium nitroprusside.

Cyanide is especially toxic to cells and organs with high metabolic requirements. Accordingly, the primary targets of cyanide toxicity in mammals are the cardiovascular system and the central nervous system. Acute cyanide intoxication induces respiratory arrest, unconsciousness, convulsions, tremor, and ultimately death. The long‐term exposure to nonlethal doses of cyanide is known to affect, among others, the endocrine system; it can also induce neurological defects and developmental/birth defects. In humans, the acute oral lethal dose of KCN is approximately 200 mg. In the gaseous form, the Immediately Dangerous to Life or Health value for inhaled hydrogen cyanide is 50 p.p.m. [11, 12, 13, 14, 15].

Hydrogen cyanide is a gas, but cyanide is also highly soluble in water and in biological fluids. Cyanide is a weak acid (pKa = 9.2). At physiological pH, approximately 98% of cyanide exists in the volatile undissociated form (HCN). The small size of this molecule, coupled with its solubility properties, allows it to rapidly cross‐mucous membranes, ensuring a rapid uptake, followed by broad tissue distribution and action [11, 12, 13, 14, 15].

The vast majority of biomedical literature focuses on cyanide’s cellular actions in an environmental toxicological context [11, 12, 13, 14, 15]. The cytotoxic effects of cyanide are primarily attributed to the inhibition of mitochondrial respiration, with cytochrome c oxidase (mitochondrial Complex IV) being cyanide’s principal target. The first part of the current article focuses on the various cytotoxic mechanisms of cyanide, in the environmental toxicology context, and also details the small body of evidence implicating the action of bacterial cyanide in a pathophysiological context. The second part of the review focuses on a new direction in cyanide research, which—in stark contrast to the environmental toxicological aspects of cyanide—implicates cyanide as an endogenous mammalian product, which, at low concentrations, serves biological regulatory roles. Overview of these recently emerging data leads to the conclusion that cyanide may be an endogenous, biological mammalian gaseous mediator, belonging to the class of gasotransmitters (a group of mediators which also includes nitric oxide [NO], carbon monoxide [CO], and hydrogen sulfide [H_2_S]).

## Cyanide, as an inhibitor of mitochondrial respiration and as a cytotoxic agent

The discovery of the cellular respiration and cytochrome ‘connection’ and the delineation of the effect of cyanide on these responses is the result of the work of many scientists since the 18th century. The Italian biologist Lazzaro Spallanzani was the first to develop the hypothesis that lung respiration is associated with tissue respiration, a concept that remains one of the milestones of modern physiology [16]. Early studies on the inhibition of cellular respiration with cyanide were performed at the beginning of the 20th century by Battelli and Stern, who, studying oxidative reactions in animal tissue extracts in the presence of oxygen, observed that this process was inhibited by cyanide. Particularly, the experiments consisted of following the catalytic conversion of ‘Nadi’ reagent (a mixture of 1‐naphthol and N,n‐dimethyl‐p‐phenylenediamine) into indophenol blue, and the enzyme responsible for this reaction was called indophenol oxidase [17]. Some years later, the pioneering investigations of Warburg led to postulate the respiration theory, according to which, during cellular respiration, molecular oxygen is complexed with a catalytic iron compound called ‘respiratory enzyme’ (*Atmungsfermentum*) [18]. This theory was supported by the observation that cell respiration was inhibited by substances known to interfere with iron‐catalyzed reactions, such as cyanide and CO. In a follow‐up study, Warburg and co‐workers, taking advantage of the photolabile bond between CO and ferrous heme, concluded that the iron of the ‘respiratory enzyme’ was complexed with a heme prosthetic group [19]. In 1929, Keilin identified this ‘respiratory enzyme’ as indophenol oxidase, later called cytochrome c oxidase (CCOx) [20]. Keilin showed that the activity of this enzyme was coupled with tissue respiration and, importantly, its activity was fully inhibited by low concentrations of cyanide [20]. In 1939, Keilin and Hartree showed that CCOx consisted of two cytochromes referred as *a* and *a_3_* and that cytochrome *a_3_* was able to combine with cyanide and CO [21]. The role of cyanide in the inhibition of CCOx was subsequently validated in *in vivo* studies, in which rats subjected to intraperitoneal injection of cyanide showed a significant decrease of CCOx activity in the brain. Treatment of cyanide correlated with an anoxic state, resulting in a shift from aerobic to anaerobic metabolism, thus supporting a model in which cyanide inactivates CCOx by interfering with the utilization of molecular oxygen [22].

In mammals, CCOx (EC1.9.3.1) is an enzymatic complex made of 13 different subunits. Among them, the subunits I (57 kDa), II (∼ 25 kDa), and III (30 kDa) are involved in proton pumping activity responsible for the generation of the mitochondrial membrane potential (ΔΨ_m_) [23, 24]. The role of the other subunits has long been considered uncertain although is now recognized that some of them play an important role in modulating CCOx’s activity. For instance, when the intramitochondrial ATP/ADP ratios are high, the energetic requirement lowers with a concomitant downregulation of the electron transport chain (ETC) activity. Interestingly, it has been shown that the subunit IV of CCOx displays a binding site for ATP and the interaction with this effector induces an allosteric inhibition of the enzymatic activity [25]. Moreover, as reviewed by Hüttemann and colleagues, CCOx contains phosphorylation sites in its different subunits (I, II, IV, Va). This post‐translational modification induces a partial inhibition of CCOx and is important for maintaining ΔΨ_m_ at ‘healthy’ levels (around 120 mV), which are sufficient for ATP synthesis [26]. Dysregulation of the CCOx phosphorylation (hypo‐ or hyperphosphorylation) has been associated with bioenergetic deficits. For instance, TNF‐α‐mediated acute inflammation is accompanied by phosphorylation on Tyr304 of subunit I of CCOx, resulting in full inhibition of its enzymatic activity, eventually leading to drop of ΔΨ_m_ and ATP depletion. Conversely, dephosphorylation of CCOx leads to an increase of ΔΨ_m_ over 150 mV and may lead to ROS generation and oxidative stress, a condition typically observed during ischemia/reperfusion [26]. Similarly, S‐glutathionylation, a post‐translational modification involved in modulation of energy metabolism and oxidative damage prevention, has been observed in different mitochondrial complexes, including CCOx [27, 28]. As shown working on HepG2 cells and isolated enzyme from bovine heart, we have reported that S‐glutathionylation induces a downregulation of CCOx activity, and interestingly, this post‐translational modification could be reversed by nanomolar concentrations of cyanide [29]. As discussed below, CCOx is also targeted by NO which is responsible for partial inhibition of its enzymatic activity and maintaining ΔΨ_m_ levels under control [30].

**Fig. 1:**
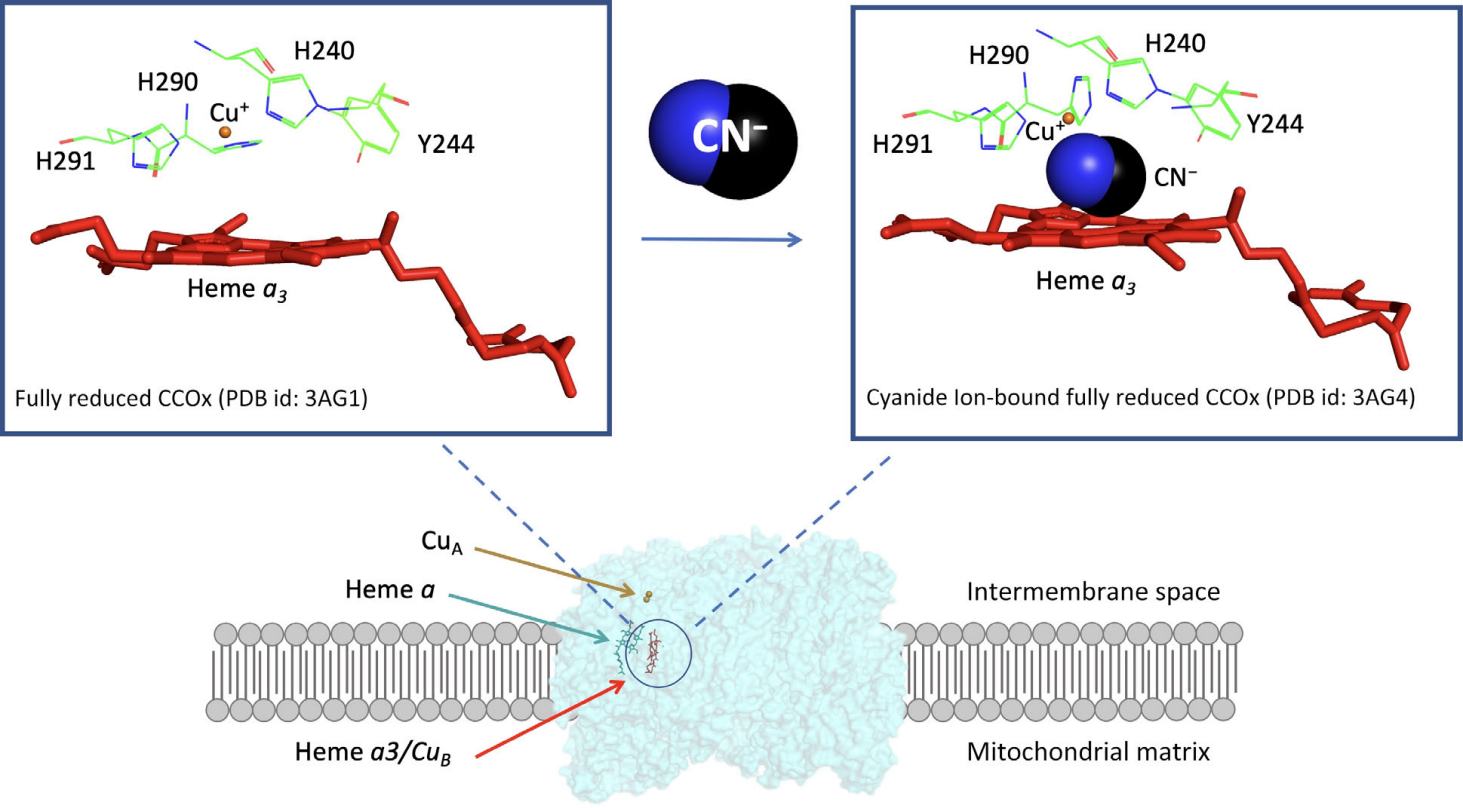
Cyanide as a Complex IV inhibitor and mitochondrial poison. CCOx dimer bounded to CN^‐^. CCOx is localized in the mitochondrial inner membrane with the cytochrome c binding site exposed to the intermembrane space. The redox centers, namely, Cu_A_, heme *a,* and the binuclear center heme *a_3_*/Cu_B_ of a single monomeric unit are represented in different colors. In the reduced state, Cu_B_ is coordinated by His290, His291, His240, and Y244, and upon binding of cyanide, His290 is displaced, thus allowing the accommodation of cyanide between Cu_B_ and heme *a_3_*. From the Protein Data Bank coordinates of the fully reduced bovine heart CCOx in the presence and absence of cyanide (PDB Id: 3AG1 and 3AG4), deposited by Muramoto and colleagues [30, 36].

For biochemical and biophysical studies, CCOx is typically obtained from beef heart and exists in homo‐dimeric form. The single monomeric functional unit harbors 4 redox centers consisting of a bimetallic Cu_A_, heme *a,* and the O_2_‐binding catalytic site made of heme *a_3_* coupled with Cu_B_ (Fig. 1) [23]. The first electron acceptor is Cu_A_ is localized in subunit II and is exposed to mitochondrial intermembrane space, where it interacts with the electron donor cytochrome c, thus undergoing one‐electron reduction. Subsequently, reduced Cu_A_ donates the electron to heme *a*, thus triggering a structural transition involved in proton pumping [31]. Eventually, electrons are transferred from heme *a* to the heme *a_3_*/Cu_B_ binuclear redox center. The latter is located in subunit I and represents the catalytical site where the reduction of oxygen takes place [32]. *A_3_*/Cu_B_ is also targeted by a range of small molecules that are known to inhibit CCOx activity, namely, cyanide, H_2_S, CO, and NO [26].

Mechanistically, the first evidence of the binding of cyanide to CCOx was reported by Keilin, who showed that cyanide binds to the heme *a_3_*, both in the oxidized (*a_3_^3+^*) and reduced (*a_3_^2+^*) state. Particularly, the complex with the *a_3_^3+^* seems to be more stable than the one with *a_3_^2+^*, since the latter state is prone to auto‐oxidation [21]. However, the interaction between *a_3_^3+^* and cyanide has been reported to be too slow to have any physiological relevance, thus suggesting that the *a_3_^2+^* state of the enzyme is the actual binding form [33]. A subsequent study comparing the binding parameters of cyanide to different states of CCOx, estimated that the partially reduced enzyme, populated during turnover, displays a binding affinity to cyanide that is 5 orders of magnitude higher as compared to the fully oxidized enzyme [34]. As shown by sequential mixing and rapid scan stopped flow spectroscopy, a one‐electron reduction of *a_3_*/Cu_B_ is sufficient to induce a rapid cyanide binding [29]. According to this model, the very first binding site is Cu_B_ and two possible mechanisms are conceivable. A single electron transfer from Cu_A_/cytochrome *a* to *a_3_*/Cu_B_ (a) induces a structural transition in the binuclear *a_3_*/Cu_B_ center or, (b) reduces Cu_B_, which is the site with the highest redox potential, from Cu^2+^ to Cu^1+^. Both mechanisms lead to the dissociation of Cu_B_ from a bound intrinsic ligand (possibly His290), thus allowing the binding to cyanide [35, 36]. Cyanide toxicity is generally considered to be due to an irreversible inhibition of the binding of molecular oxygen to the binuclear site *a_3_*/Cu_B_, although some reports showed that cyanide‐induced inactivation of CCOx can, in fact, be also reversible. Intriguingly, Pearce *et al*. reported that cyanide inhibition of CCOx can be partially reverted by NO, another CCOx inhibitor (∼ 10^3^‐fold more effective than cyanide, as shown in Table 1) [34, 37, 38, 39]. Importantly, NO proved to displace cyanide and, when the enzyme was in turnover, NO is gradually degraded (oxidized) in solution [39, 40]. Moreover, as shown in freshly isolated rat liver mitochondria or hepatocytes, the inhibition of oxygen consumption and ATP synthesis after 10‐min incubation with 250 µm of KCN was fully reversed by extensive washing in the incubation medium [41, 42]. The reversibility of the cyanide inhibition of CCOx may open the possibility that cyanide, similarly to NO, plays a physiological role in the modulation the cell respiration.

**Table 1:** Reaction characteristics of NO, CO, H_2_S and HCN with cytochrome c oxidase

Inhibitor	Enzyme form	K_on_	K_off_	K_off_/K_on_	References
		(m ^−1^ s^‐1^)	(s^‐1^)		
NO	Reduced	1.5 × 10^8^	4 × 10^−3^	0.03 nm	[23]
CO	Reduced	8 × 10^4^	0.023	0.29 µm	[37]
H_2_S	Reduced	1.5 × 10^4^	6 × 10^−4^	0.04 µm	[38]
HCN	Reduced, in turnover	2 × 10^6^	4.7 × 10^−2^	0.02 µm	[34]

Since oxidative phosphorylation is essential for ATP generation in mammalian cells, a cyanide‐mediated shutdown of Complex IV activity, and a consequent inhibition of mitochondrial electron transport and aerobic ATP generation would be expected to severely affect the function of all cells, especially those with high bioenergetic demand (e.g., developing/proliferating cells, neurons, cardiac myocytes). Indeed, *in vivo* or *ex vivo* studies show that ATP and high‐energy phosphate content of the tissues decrease rapidly and precipitously after systemic exposure to cyanide [43, 44, 45, 46, 47, 48, 49] and the inhibition of mitochondrial Complex IV is well detectable in various tissues *ex* *vivo* [22, 48, 50, 51, 52]. In line with the inhibition of mitochondrial function, tissues exposed to cyanide lose their ability to extract oxygen, even in the presence of adequate blood flow [53, 54], a phenomenon called ‘cytotoxic hypoxia’. Nevertheless, in tissues with high levels of phosphocreatine pools (e.g., heart and skeletal muscle), the energetic deficit manifests itself in phosphocreatine depletion, rather than ATP depletion; the decrease of mitochondrial ATP generation may be temporarily compensated by a backward flux of creatine kinase [44, 46, 49, 55].

Similarly to the *in vivo* findings, a large number of *in vitro* studies demonstrate that cyanide (typically applied in the concentration range of 100 µm–3 mm) produces a significant decrease in cell viability and/or cell proliferation, and these effects are usually linked to inhibition of cytochrome c oxidase activity and a rapid decrease in intracellular ATP content [42, 56, 57, 58, 59, 60, 61, 62, 63].

However, in addition to the effects of cyanide on mitochondrial Complex IV—a variety of additional, deleterious effects of cyanide have been reported in various *in vitro* experimental systems. For example, cyanide (at the relatively low concentration of 50–100 µm) was shown to enhance NMDA receptor activation and the consequent calcium mobilization response [64, 65]. Cyanide, at millimolar concentrations, was reported to exert selective effects on NMDA receptor subtypes: It potentiates the activation of NR1/NR2A receptors, most likely via the chemical reduction of NR2A, but it does not potentiate the activation NR1/NR2B receptors (rather, it exerts a slight inhibitory effect) [66, 67]. Exposure of various cell types to cyanide was also found to produce an intracellular Ca^2+^ response, which, at low cyanide concentrations (starting at 1 µm) produces a stimulation of intracellular inositol triphosphate level, followed by Ca^2+^ mobilization from intracellular calcium pools [64]. At higher cyanide concentration, (starting at 100 µm and becoming pronounced at 300 µm–1 mm) Ca^2+^ mobilization in various neuronal cell types was also shown to involve the influx of extracellular calcium through L‐type membrane calcium channels and NMDA receptors as well as further intracellular Ca^2+^‐release from the endoplasmic reticulum and mitochondria [65, 68, 69, 70, 71, 72, 73, 74, 75] as well as an inhibition of active Ca^2+^ extrusion from the cells [70]. Increased intracellular Ca^2+^, in turn, increases the activity of Ca^2+^‐dependent enzymes (e.g., phospholipase A2, caspases, proteases, and endonucleases) [76, 77, 78, 79, 80, 81]. Cyanide (in millimolar concentrations) induces nuclear and mitochondrial DNA fragmentation [82, 83]; it can also cause microtubular disruption [84]. Cyanide treatment of various cell types has also been shown to suppress K_ATP_ channel and inwardly rectifying K^+^ channel activity [85, 86]. Activation of NF‐κB after cyanide exposure [87, 88] may link cyanide’s cellular effects to changes in signal transduction, gene expression and/or to regulated pathways of cell death (apoptosis).

In neurons, the apoptotic form of cyanide‐induced cell death—which typically occurs at approximately 100–300 µm cyanide—appears to be more related to calcium overload, BNIP3 mobilization, cytochrome c release, and caspase activation, while the necrotic form of cell death (which typically occurs at cyanide concentrations higher than 300 µm) is linked to mitochondrial inhibition of ATP generation, mitochondrial dysfunction (which also involves upregulation of uncoupling protein 2 in the mitochondria), activation of poly(ADP‐ribose) polymerase (PARP), and overall cellular bioenergetic deficit [89, 90, 91]. In the brain, cyanide was also demonstrated to cause a rapid‐onset increase in lipid peroxidation [92, 93, 94]. High concentrations of cyanide (1–10 mm) cause a significant intracellular acidification as well [95]. Cyanide, via activating the above‐listed pathophysiological pathways, can induce the release of various neurotransmitters (e.g., glutamate and dopamine) from the damaged cells [92, 96]. At extremely high concentrations, cyanide can also exacerbate the neurotoxic effect of various mediators, for example, NMDA and dopamine [97, 98, 99, 100, 101]. These actions may contribute to cyanide‐associated CNS injury *in vivo*.

**Fig. 2:**
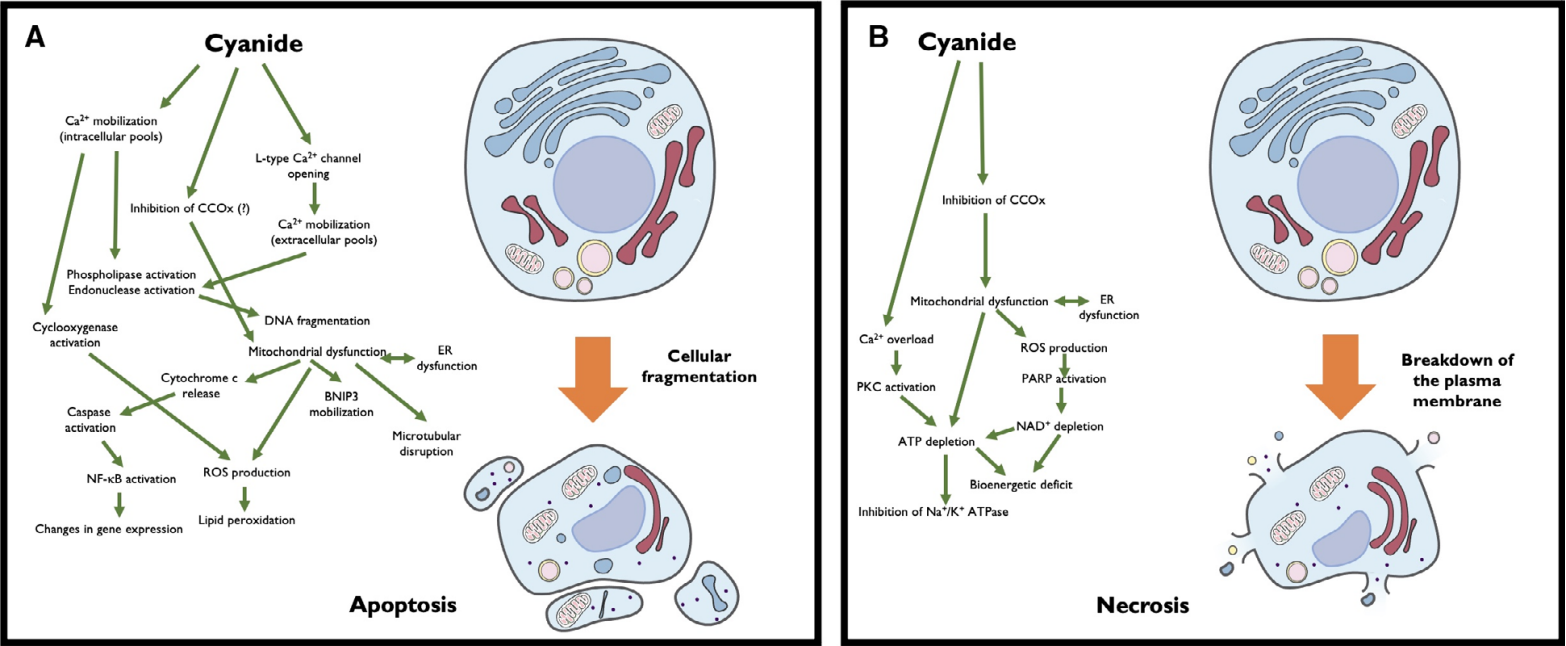
Pathways contributing to the cytotoxic actions of cyanide in mammalian cells. (A) Typically, at high micromolar concentrations (e.g., 100–300 µm in neurons), cyanide induces apoptotic cell death. Early stages of this process include mobilization of calcium from intra‐ and extracellular pools. This calcium mobilization (possibly, in combination with a partial inhibition of CCOx and mitochondrial dysfunction, coupled with ER dysfunction), stimulates various effectors of apoptotic cell death. For instance, ROS are generated either by cyclooxygenase (which is stimulated by calcium mobilization) or by the mitochondria (as a consequence of CCOx inhibition). ROS and calcium stimulate various apoptotic effectors (e.g., endonucleases, caspases), BNIP3 (BCL2 and adenovirus E1B 19‐kDa‐interacting protein 3) and signaling pathways (e.g., NF‐κB). These processes culminate in apoptotic cell death; these cells are typically eliminated by phagocytes and do not exacerbate local inflammatory responses. (B) Typically, at low millimolar concentrations (e.g., 1–3 mm), cyanide induces necrotic cell death. A central part of this process is a pronounced inhibition of CCOx and mitochondrial dysfunction, also reflected in severe degree of cellular ATP depletion. An additional factor in this process is calcium overload, followed by activation of PKC. Mitochondrially derived ROS (perhaps together with ROS formed by other cellular sources and perhaps also in combination with NO to form peroxynitrite) induces DNA single strand breakage, which is a direct activator of the nuclear enzyme PARP. PKC activation, and PARP activation, further depletes cellular NAD^+^ and ATP levels. Because of the low cellular ATP, cells are unable to maintain the activity of membrane pumps and the membrane potential dissipates and the cell starts to ‘leak’ and release its intracellular content. During full‐fledged necrosis, all cellular content is released as the cell disintegrates. This process can, in turn, lead to additional local or remote inflammation.

Some of the effects of cyanide listed in the previous paragraph may be secondary to inhibition of Complex IV. For instance, when inhibition of mitochondrial electron transport occurs, the percentage of oxygen incompletely reduced by mitochondria will be increased, leading to oxygen free radical (ROS) formation due to the dysfunctional operation of the electron transport chain [89, 99, 100, 102, 103]. ROS, in turn, can promote the activation of NF‐κB after cyanide exposure [87]. ROS may also induce the release of calcium from the mitochondria, which, in turn, may activate various intracellular enzymes that mediate DNA fragmentation or apoptosis [82, 104, 105, 106]. Microtubular assembly and movement is also a highly ATP‐dependent process, and its disruption would be expected if mitochondrial ATP generation is impaired. The effects of cyanide on various potassium channels may also be—partially or completely—the consequence of intracellular ATP suppression, via processes that have been proposed to involve, in some cases, modulation of phosphatase activity [85, 86]. However, some of the other reported cellular effects of cyanide are probably independent of Complex IV inhibition (or ATP depletion or ROS generation). For example, the stimulatory action of cyanide on NMDA receptors is independent of mitochondrial or bioenergetic actions (see below). Some of the key pathways of cyanide cytotoxicity, leading either to apoptotic, or necrotic cell death, are shown in Fig. 2.

There are several lines of studies that are inconsistent with the ‘central dogma’ that cyanide causes an irreversible inhibition of Complex IV activity, followed by the loss of ATP, and this effect is directly responsible for the impairment of cell viability and function. For instance, in hepatocytes, the cyanide (250 µm–1 mm, 10 min) induced a depletion of cellular ATP pools, ATP levels rapidly and completely recovered after washout of cyanide from the medium [42]; this response pattern is not consistent with an irreversible inhibition of ATP biosynthesis. A similar reversibility of the cyanide‐induced suppression of ATP was also noted earlier in a perfused liver preparation [107]. It is possible that, in the presence of a sufficient concentration of reducing equivalents, cyanide may be physiologically dissociated from the CCOx/inhibitor complex: that is, inhibition of CCOx by cyanide, at least under certain conditions and in certain cell types, can be reactivatable. (A similar type of reactivation, using various pharmacological approaches, is one of the principles of cyanide antidotes [108]). Moreover, as noted above, cyanide already at relatively low concentrations (e.g., 50 µm) enhances NMDA receptor activation. This response has been demonstrated in reductionist preparations that don’t contain mitochondria (e.g., measurement of single channel activity in excised outside‐out membrane patches) and therefore must be the consequence of direct effects of cyanide on these receptors, possibly via a direct action on redox sensitive disulfide groups [66, 67]. At higher concentrations [1 mm], cyanide may also, at least in part, act in a direct fashion on the NMDA receptor, by interacting with its Mg^2+^ block [98]. Sun and Reis have reported that cyanide (30–300 µm) activated Ca^2+^ currents in pacemaker neurons of rat rostral ventrolateral medulla *in vitro* in the absence of significant cellular damage [109]. A similar, ATP‐independent suppression of myocardial contractility has also been reported [110]. Moreover, in a guinea pig hippocampal preparation, cyanide, at the relatively low concentration range of 10 µm–200 µm, decreased synaptic transmission; the effects were rapidly reversible by washout and therefore are unlikely to be related to an irreversible inhibition of Complex IV [111]. In a rat diaphragm preparation, cyanide (100 µm–1 mm), unexpectedly, produced an initial increase (rather than decrease) of muscle contractility, followed by a decrease at later time points; neither the increase nor the decrease in contractility was associated with any detectable change in the ATP content of the muscle (although the decline of contractility was associated with decreased phosphocreatine levels); moreover, contractile function returned to normal after washout of cyanide from the preparation [112]. The effect of cyanide was suggested to be related to an action of the contractile apparatus, perhaps an ability of cyanide to influence the sensitivity of the contractile proteins to Ca^2+^ [112].

A separate class of cyanide’s cellular actions relates to the activation of protein kinase C (including the PKCɛ isoform), with a variety of signaling and/or deleterious responses (including the phosphorylation of α1c subunit of L‐type Ca^2+^ channel and the α2 subunit of Na^+^‐K^+^‐ATPase). These effects can be relevant in the context of cyanide toxicity, but they do not appear to be associated with a detectable decrease in total cellular ATP levels [113]. Importantly, the observation that PKC inhibition can attenuate the cyanide‐induced cell necrosis *in vitro* and *in vivo* [49, 114] indicates that the PKC‐dependent effects may be independent from the mitochondrial/bioenergetic responses induced by cyanide; in neuronal tissue they may be, however, the consequences of NMDA receptor activation [115].

It should be mentioned that cyanide is a direct inhibitor of several enzymes in addition to cytochrome c oxidase. These include succinic acid dehydrogenase, superoxide dismutase, carbonic anhydrase, glutathione peroxidase, NADPH oxidase, myeloperoxidase, glutamic acid decarboxylase, and gamma‐aminobutyric acid transaminase [115, 116, 117, 118, 119, 120]. In contrast, monoamine oxidase A (but not monoamine oxidase B) activity was reported to increase in response to cyanide [121]. The effect of cyanide on NADPH oxidase is variable; inhibition, activation, or no effect were all reported under various experimental conditions [122, 123, 124, 125]. The potential contribution of cyanide’s action on these enzymes is incompletely understood but is most likely only relevant in the toxicological context. In Syrian hamster embryo cells, the cyanide (500 µm) induced DNA fragmentation and cytotoxicity appears to be a combined result of mitochondrial inhibition, mitochondrial ROS release as well as a parallel, independent inhibition of catalase and superoxide dismutase [126].

It should be emphasized that *in vitro*, the effect of cyanide on mitochondrial function and cell viability can be variable, depending on the cell type, the characteristics of the culture medium used (glucose, CO_2_, pH), as well as different physicochemical factors (e.g., the degree of loss of cyanide gas from the culture medium due to outgassing) [63, 127]. Also, cells in culture can be highly glycolytic. Thus, the cyanide‐induced shutdown of aerobic ATP generation may be partially compensated by ATP generation from glycolysis [22, 128, 129, 130, 131, 132, 133]. In fact, in many experiments, complete inhibition of cellular ATP synthesis can be only achieved when the cyanide‐mediated inhibition of oxidative phosphorylation is combined with pharmacological inhibitors of glycolysis [29, 129, 134, 135]. However, cellular ATP generation after cyanide exposure can also be influenced by the fact that cyanide may also have direct inhibitory effect on glycolysis: in hepatocyte intermediary metabolism, cyanide was reported to decrease glucose catabolism by inhibiting the glycolytic pathway (while also producing a shunt, with a resulting increase in glucose catabolism by the pentose phosphate pathway) [136].

## Bacterial cyanide can exert pathophysiological effects to mammalian cells

In the previous sections, the mammalian cell was viewed as a target of environmental cyanide exposure. There are, however, some pathophysiological conditions where bacterially generated cyanide has been implicated as a pathophysiological (cytotoxic) factor to mammalian cells. The best studied example is cystic fibrosis (CF), a progressive genetic disease, in which the colonization of the lung with *Pseudomonas aeruginosa* (an opportunistic pathogen) is a common occurrence and a significant pathogenetic factor [137, 138, 139]. As noted earlier, *Pseudomonas aeruginosa* is a cyanide‐producing bacterium; cyanide concentrations in the supernatant of these bacteria can reach several hundred micromolar levels [140, 141, 142]. It appears that this bacterially produced cyanide can exert deleterious effects on the host lung. Indeed, cyanide levels as high as several hundreds of µm have been detected in the sputum of CF patients with *Pseudomonas aeruginosa* lung infection [142, 143, 144]; secondary species such as cyanogen chloride (CNCl) and the carbamoylating species cyanate (OCN^−^) were also detected [145]. HCN gas has also been detected in the exhaled breath of these patients; in fact, exhaled HCN has been proposed as a potential diagnostic biomarker of *Pseudomonas* colonization of the lung [146, 147, 148, 149, 150, 151].

*Pseudomonas*‐derived cyanide serves as a bacterial ‘weapon’ to suppress the growth of competing, co‐colonizing bacteria (e.g., *Staphylococci* or *Burkholderia cenocepacia*) in the lung [152, 153]. Moreover, *Pseudomonas*‐derived cyanide can have deleterious effects on the host airway. As discussed in the previous section, cyanide concentrations of 100 µm and higher are significantly cytostatic or cytotoxic to mammalian cells. Therefore, it is likely, that the pulmonary epithelial cells, which are exposed to the highest local concentrations of cyanide, will be adversely affected by cyanide. Indeed, exposure of human pulmonary epithelial cells (obtained from nasal brushings) to cyanide (75–150 µm) produced a significant decrease in ciliary beat frequency, which may predict that *Pseudomonas* in CF lungs may impair the ability of the epithelial cell to perform effective mucociliary clearance [154]. Moreover, bacterial supernatant transfer studies demonstrate that concentrated supernatants of *Pseudomonas aeruginosa* can suppress mitochondrial respiration via inhibition of Complex IV activity in mammalian cells; this suppression is no longer noted after the deletion of the cyanide‐producing enzyme in the bacteria [29]. As discussed by Anderson [143], bacterial cyanide may also have additional adverse effects on the host beyond inhibiting mitochondrial function and viability of epithelial cells, such as immunosuppressive effects and/or interference with the function of neutrophil granulocytes. Taken together, bacterial cyanide may well be a significant contributor to the pathogenesis of cystic fibrosis‐associated pulmonary dysfunction. Importantly, Ryall *et al*. demonstrated that sputum cyanide shows a negative correlation with lung function in CF patients [155], perhaps indicating that cyanide contributes to lung dysfunction in CF (although this may also be related to the fact that patients with more severe *Pseudomonas* colonization tend to have a more advanced lung disease, in general). To our knowledge, so far there have not been any experimental or clinical studies performed attempting to scavenge or neutralize or inactivate the cyanide‐producing gene in *Pseudomonas* in the context of experimental CF therapy. There are several classes of cyanide scavengers/antidotes, and some of these agents—for example, vitamin B12a—tend to have a fairly excellent safety profile and are already used in clinical trials, for instance in the context of burn‐associated cyanide poisoning [156]. Also, inhaled NO has been proposed to interfere with the cyanide‐producing ability of *Pseudomonas* [157]. Approaches exploiting these mechanisms may be the topics of potential future preclinical proof‐of concept studies and possibly subsequent clinical trials.

There are many unexplored directions in the above line of research. For instance, the lung can also be colonized by cyanide‐producing bacteria in other forms of pulmonary disease (e.g., noncystic fibrosis bronchiectasis and asthma) [138]. It should be also noted that endogenous (mammalian cell produced) cyanide may also be relevant factor in contributing to sputum or exhaled air cyanide levels [145, 158]. Although the focus of most work related to bacterial cyanide production focuses on *Pseudomonas*, several other bacterial pathogens (e.g., *Burkholderia cenocepacia*) also produce cyanide. Some of these pathogens can colonize the lung in CF [159]; others may be pathogenetic factors in other lung diseases (e.g., ‘cepacia syndrome’ or necrotizing pneumonia) or in systemic infections and septic shock. Indeed, when various ‘model organisms’ are co‐cultured with cyanide‐producing *Pseudomonas* species, the model organisms exhibit signs of metabolic suppression and injury. For instance, co‐culture of *C. elegans* with cyanide‐producing *Pseudomonas* produces rapid paralysis and killing of the nematodes, with cyanide being the principal agent responsible for this action [160, 161]. Similarly, cyanide‐producing human *Pseudomonas* isolates are toxic, in a significant part due to cyanide production, to *Drosophila melanogaster* co‐cultures [162]. *Pseudomonas* species are common pathogens in urinary tract infections; the potential role of the ‘cyanide connection’ in this context remains to be explored.

Population‐based incidence studies indicate that only approximately 2–8% of all septic shock cases include systemic *Pseudomonas* bacteremia, but these cases tend to be associated with severe clinical picture and high mortality [163, 164, 165, 166, 167]. To our knowledge, circulating cyanide levels have not been assessed in these patients. It is conceivable that systemic cyanide may impair mitochondrial function in *Pseudomonas* bacteremia via the mechanisms discussed above. Based on various vascular studies with cyanide [168, 169, 170, 171, 172], we believe it is also conceivable that circulating cyanide in *Pseudomonas* sepsis may also exert deleterious effects on the endothelial cells and vascular smooth muscle cells, impairing endothelial function and contributing to vascular contractility. Further work is required to investigate whether systemic *Pseudomonas* infection, and the consequent systemic cyanide exposure may contribute to the pathogenesis of cell and organ dysfunction.

## Endogenous generation of cyanide in mammalian cells

As discussed in the Introduction section, many living organisms contain cyanide‐producing enzymes; there are many strains of cyanide‐generating bacteria, which produce cyanide for regulatory purposes or as a virulence factor; moreover, plant‐generated cyanide mediates various processes, from plant growth to plant immunity. Although cyanide synthesis by mammalian cells has not been investigated extensively, several studies indicate that mammals have detectable circulating cyanide concentrations, and cyanide production has been demonstrated in various mammalian cells and tissues. Utilizing a variety of methods the basal circulating cyanide concentration in healthy volunteers has been estimated to be in the range of 1–5 µm [173, 174, 175, 176, 177]. Perhaps these methods overestimate the concentration of cyanide, at least in part because some of these methods may also include thiocyanide. Using a capillary gas chromatography method, basal cyanide concentrations in healthy humans were found to be lower: approximately 300 nm [178]. The cyanide metabolite 2‐aminothiazoline‐4‐carboxylic acid (ATCA) is also detectable in significant levels in the blood and tissues of humans or experimental animals without any prior exposure to exogenous source of cyanide [175, 179, 180]; another cyanide reaction product (protein‐bound thiocyanate) was also detected (in approx. 600 nm concentration) in the blood of healthy individuals [181]. Cyanide levels in the exhaled breath of healthy human beings have been quantified to be in the 4–62 ppb range [173, 182, 183, 184], with significant increases in patients with *Pseudomonas* colonization in their lungs (see above). Circulating cyanide levels are increased 3‐ to 4‐fold in smokers; they are markedly increased in various exogenous cyanide exposure situations (for instance, blood cyanide levels increasing to approximately 25–100 µm in fire victims) [175, 178, 185]. Interestingly, circulating cyanide and cyanide metabolites also exhibit significant increases in hemodialysis patients, most likely due to impairment of various cyanide detoxification and elimination pathways [186, 187, 188].

Through the use of enzyme repositories systems, such as BRENDA (https://www.brenda‐enzymes.org/), we were able to find at least 14 different enzymes producing cyanide, but not all of them are relevant for the scope of the present review. Indeed, many of them are expressed in plants as part of chemical defense system adopted in case of peril, represented by pathogens or herbivores. For instance, many plants biosynthesize cyanogenic compounds, such as cyanohydrins, to be used as substrates for the rapid production of cyanide [189]. At least 5 cyanide‐producing enzymes have been identified in mammals. Within this subgroup, a distinction must be made between enzymes producing cyanide from *endogenous* substrates vs. those producing cyanide from xenobiotics. Indeed, while the former class of enzymes are potentially capable of releasing cyanide constantly (to yield cyanide in low and nontoxic concentrations), the latter class of enzymes release cyanide only in certain circumstances, such as upon exposure to cyanogenic compounds derived from the diet (fruit and vegetables) or after exposure to drugs or pesticides.

From currently known list of mammalian enzymes producing cyanide from endogenous substrates, myeloperoxidase (MPO) [EC1.11.2.2] is potentially the most significant one. MPO is a heme‐containing enzyme, encoded in humans by the *MPO* gene (NCBI gene ID: 4353) located on chromosome 17. MPO is abundantly expressed in neutrophils and monocytes (but also present in many other cell types), and it is believed to play an important role in the innate immune system [190]. This enzyme has been shown to display a substrate promiscuity, being able to catalyze a number of side reactions [191], including the generation of cyanide from glycine [192, 193]. From the mechanistic point of view, MPO is involved in the process responsible for the protection against microbes’ infections by releasing toxic agents, such as ROS [194]. The most widely recognized activity of MPO is the oxidation of chloride, bromide, or thiocyanate to the strongly oxidizing agents, HOCl, HOBr, and HOSCN, respectively. For these reactions, the enzyme uses hydrogen peroxide (H_2_O_2_) (released from superoxide dismutase). These activities are controlled by environmental factors, such as substrates availability and pH. For instance, although thiocyanate has a higher affinity for MPO with respect to the other substrates, the reaction with chloride is often favored because of the higher abundance of this anion in the cellular milieu [191]. The pH also plays a crucial role, as it influences the affinity of both H_2_O_2_ and Cl^‐^ for MPO. Particularly, H_2_O_2_ ‐dependent reactions occur preferentially at pH values around 6, while the *K*_M_ of Cl^‐^ has been shown to be optimal at lower pH. As a consequence, depending on the pH, other ligand may compete with the ‘canonical’ substrates for the enzymatic active site. This has been reported, for instance, for glycine, which at low pH undergoes catalytic chlorination by MPO to generate N‐monochloro‐glycine intermediate, followed by nonenzymatic release of cyanide [192]. Conversely, at higher pH values, cyanide has been reported to be chlorinated to cyanogen chloride (ClCN) by MPO, thus suggesting a dual role for cyanide, being both a substrate and a product of MPO [192, 193].

Another mammalian enzyme capable of cyanide generation is cyanocobalamin reductase [EC1.16.1.6], also known as CblC or MMACHC [195]. This enzyme is generally known for its role in the metabolism of vitamin B_12_. Vitamin B_12_ is commonly used in multivitamin formulations in the form of cyanocobalamin (CNCbl), which is converted in the body to its active form methylcobalamin (MeCbl) and 5’‐deoxyadenosylcobalamin (AdoCbl) [195]. This reaction consists of a reduction CNCbl, in the presence of reducing equivalents provided by NADPH and FADH_2_, leading eventually to the reduction of Co^+3^ to Co^+2^ and the concomitant release of cyanide [195]. In humans, cyanocobalamin reductase is encoded by the *MMACHC* gene (methylmalonic aciduria and homocystinuria type C protein), located in chromosome 1 (NCBI gene ID: 25974). Mutations of this enzyme have been associated with methylmalonic acidemia, an inborn metabolic disorder leading to neuronal damage [196, 197].

Another cyanide‐producing mammalian protein has been reported to be hemoglobin, which catalyzes the peroxidation of thiocyanide thus generating cyanide and sulfate as final products [198]. This reaction has been shown to occur only in the erythrocytes, where the enzymes catabolizing cyanide are absent. The physiological relevance of this reaction has been shown both *in vitro* and *in vivo* systems, thus suggesting the existence of a metabolic equilibrium between thiocyanate and cyanide [198, 199].

**Fig. 3:**
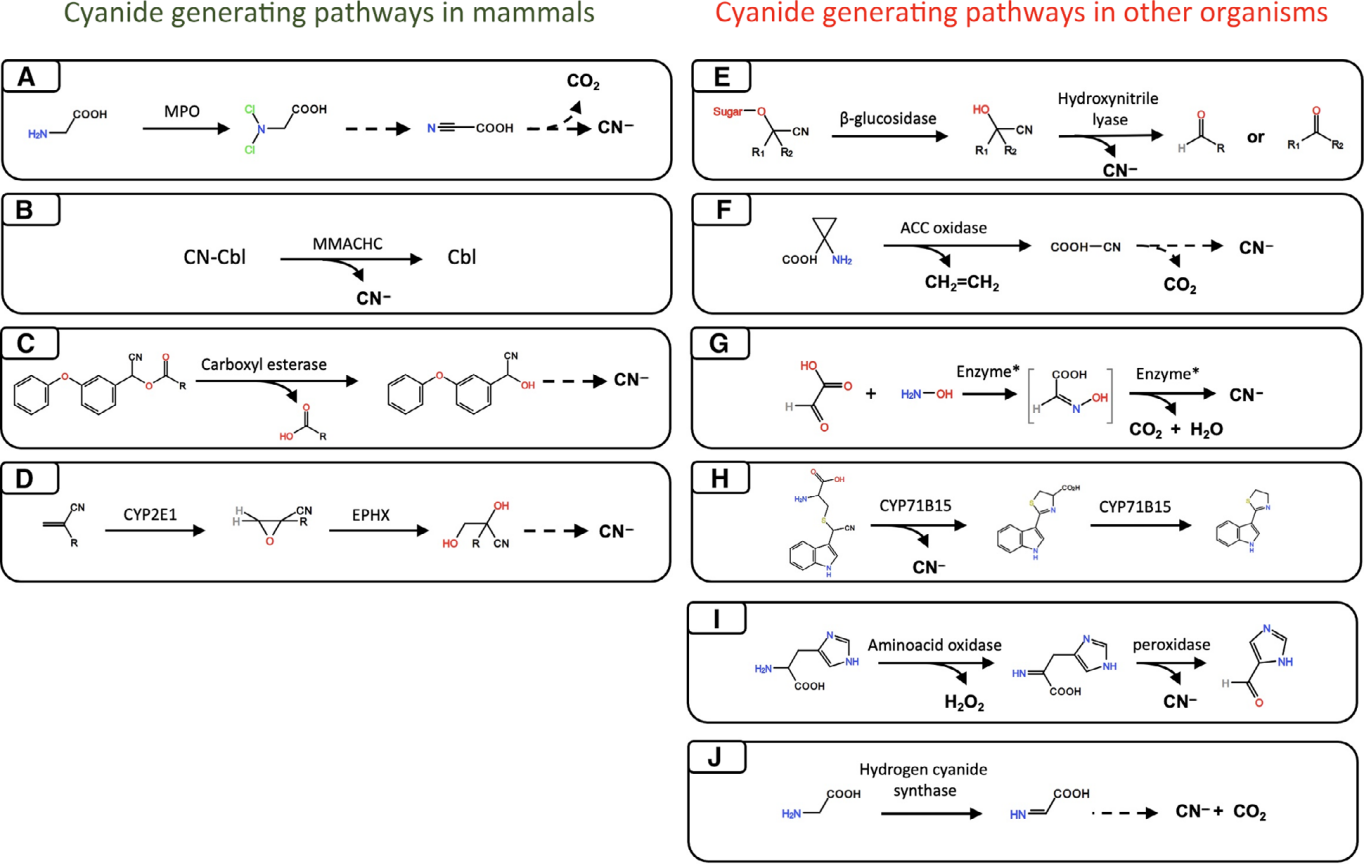
Physiological cyanide‐generating systems in mammals and other organisms. Solid arrows represent enzyme catalyzed reactions, while dashed arrows stand for nonenzymatic degradation. (A) MPO catalyzes the chlorination of glycine into N‐dichloro‐glycine, which is an unstable compound and decomposes to its corresponding nitrile, followed by nonenzymatic release of cyanide and carbon dioxide. (B) Cyanocobalamin reductase (MMACHC) catalyzes the decyanation of CNCbl to yield cob(II)alamin (Cbl) and cyanide. (C) Carboxyl esterase catalyzes cypermethrin (an insecticide of the family of pyrethroids) hydrolysis to its corresponding cyanohydrin. Cyanohydrins are unstable species which give cyanide as product of degradation. (D) Aliphatic nitriles undergo epoxidation catalyzed by the microsomal enzyme CYP2E1, followed by liberation of cyanide by epoxide hydrolyze (EPHX). (E) In plants β‐glucosidase/hydroxynitrile lyase system is believed to be one of the main sources of free cyanide. Cyanogenic glucosides are processed by β‐glucosidase thus producing the correspondent cyanohydrin, which are then converted to aldehyde (or ketone) by hydroxynitrile lyase, with the concomitant elimination of cyanide. (F) Ethylene in plants is considered a hormone involved in many processes. The ethylene synthesis is accomplished by the oxidation of 1‐aminocyclopropane‐1‐carboxylic acid (ACC), by ACC oxidase, into ethylene and cyanoformic acid. The latter spontaneously decomposes in cyanide and carbon dioxide. (G) Cyanide production from glyoxylate has been observed in algae (*Chlorella vulgaris*), spinach (*Spinacia oleracea*), corn (*Zea mays*), and barley leaves. In the presence of hydroxylamine, glyoxylate generates glyoxylate oxime, followed by degradation into cyanide, carbon dioxide, and water. The enzyme catalyzing this reaction has a molecular mass of 40 kDa and has been established to require for its catalytic activity ADP and Mn^2+^. (H) Camalexin is a characteristic alkaloid of *Arabidopsis thaliana* accumulated upon infection of a variety of pathogens. The final steps of its biosynthesis are controlled by CYP71B15, which catalyzes both the formation of thiazolidine ring of cysteine‐indole‐3‐acetonitrile, with the concomitant release of cyanide, and the subsequent oxidative decarboxylation of dihydrocamalexic acid to camalexin. (I) Cyanide biosynthesis from aromatic amino acids (histidine, tyrosine, and phenylalanine) has been observed in the alga *Chlorella vulgaris* and in spinach leaves. The reaction has been shown to be catalyzed by amino acid oxidase co‐incubated with Mn^2+^ and horseradish‐peroxidase, thus causing the formation of cyanide. Several bacterial species (the most extensively characterized being *Pseudomonas aeruginosa*) are known to produce cyanide. The immediate precursor of cyanide is glycine which is converted to cyanide and carbon dioxide in a reaction catalyzed by hydrogen cyanide synthase.

Among the additional, currently known, mammalian cyanide‐producing enzymes, many are microsomal enzymes involved in the metabolism of cyanogenic xenobiotics. For instance, carboxylic esterase has been shown to catalyze the hydrolysis of pyrethroids, a class of insecticides, to the correspondent cyanohydrin, followed by nonenzymatic hydrolysis to an aldehyde with the elimination of cyanide [200]. Aliphatic nitriles, another class of cyanogenic compounds widely employed in the production of vitamins and pharmaceuticals, are mainly metabolized by the CYP2E1/epoxide hydrolase system. Particularly, these compounds undergo epoxidation by cytochrome CYP2E1, followed by hydrolysis catalyzed by epoxide hydrolase, a reaction considered crucial for the liberation of cyanide [201]. It is conceivable that the CYP2E1/epoxide hydrolase system may also have endogenous mammalian substrates, but this possibility has not yet been seriously explored in the literature. Similarly, the system α‐hydroxynitrile glucoside/β‐glucosidase has been suggested to be a ‘cyanide bomb’ in plants [202]. Although β‐glucosidase is widely expressed in mammalians, cyanide release is unlikely to occur because of the absence hydroxynitrile glucoside, mostly represented in the plant kingdom. The key mammalian endogenous cyanide‐producing pathways, and, for comparison, some of the most common cyanide‐producing pathways in plants and bacteria are shown in Fig. 3.

*In vitro* studies have also demonstrated the ability of mammalian cells and tissues to produce significant amounts of cyanide, at least in part, in a regulated fashion. Indeed, since the early 1980s, it has been established that phagocytizing neutrophils can produce cyanide, in part by utilizing endogenous and in part by utilizing bacterial substrates [145, 193, 203, 204, 205, 206, 207]. Moreover, the ability of MPO to generate cyanide has also been demonstrated using the purified MPO enzyme [193, 208]. In a subsequent line of studies, Borowitz and colleagues have quantified basal cyanide generation from PC12 cells (a rat cerebellar granule cell line) as 5.5 ng·10^−6^ cells/10 min, with an approximately 3‐fold increase in response to μ‐opiate agonist stimulation; similar results were also obtained in primary rat cerebellar cells [209, 210]. Muscarinic receptor stimulation, in contrast, decreased neuronal cyanide generation [211]. Glycine also increased cyanide production in PC12 cells [210]; the underlying mechanism may be related to the previously characterized pathway whereby N‐chlorination of glycine can be catalyzed by MPO, followed by secondary reactions of N‐chloramines of glycine, which yield cyanide (as well as cyanogen chloride) (see above). A pharmacological inhibitor of myeloperoxidase decreased opiate agonist‐stimulated (but not basal) cyanide production [210]. The differential effect of this agent on basal and stimulated cyanide production may indicate different enzymatic sources for these two types of cyanide production, although this possibility remains to be further investigated.

Significant levels of cyanide were measured in rat and hamster brain microdialysates *in vivo*; in these systems, μ‐opiate agonist stimulation increased cyanide levels by about 5‐fold [209]. Borowitz and colleagues estimated whole rat brain cyanide concentration of 0.14 µg·g^−1^ wet weight (i.e., approximately 7 µm), with the hippocampus and the hypothalamus being the highest cyanide‐producing regions [209]. In a recent study, Long and colleagues used confocal imaging methods and a fluorescent cyanide probe in cultured PC12 cells and measured low, but detectable levels of cyanide under baseline conditions; in line with prior findings of the Borowitz group, the cyanide signal increased with μ‐opiate agonist to the cells; the concentrations of cyanide in these cells were estimated at 8 µm under baseline conditions and 23 µm after μ‐opiate agonist stimulation [212]. Low, but detectable basal cyanide signals were also detected in cultured neurons and the hepatoma line HepG2 [212].

## Biological reactivity of cyanide in mammalian cells

Undoubtedly, the best characterized reactivity of cyanide in biological systems is its propensity to bind transition metals, such as Fe(II), Fe(III), or Co(III), where it forms low spin complexes, reflecting its strong‐field ligand character. Indeed, its negative electric charge along with its capacity to form both σ‐ and π‐bonds, allows to stabilize transition metals in different oxidation states [213]. Its reactivity toward a wide range of metals makes cyanide a potential inhibitor of many metalloproteins, thus displaying an effect which goes well beyond the mere inactivation of the oxidative phosphorylation, as previously discussed. Along with the aforementioned CCOx, the interaction of cyanide with other heme‐proteins such as hemoglobin (and myoglobin) has been extensively studied since the beginning of the last century, as reviewed by Fanelli and colleagues [214]. According to their proposed model, two molecules of cyanide are complexed in one heme moiety, thus obtaining a mono‐ and a di‐cyanide ferroprotoporphyrin IX, in a stepwise manner [214].

Another iron heme protein targeted by cyanide is MPO, whose catalytic iron is responsible for the interaction with nucleophilic substrates. As mentioned above, this enzyme both synthesizes and catabolizes cyanide; high cyanide concentrations have been reported to inhibit its enzymatic activity [119, 215]. Iron‐cyanide interactions has been reported also for nonheme iron proteins, such as transferrin and Fe‐superoxide reductase (Fe‐SOD) [216, 217]. Interestingly, cyanide has been shown to inhibit also Zn, Cu‐superoxide dismutase (Zn, Cu‐SOD): as shown with NMR spectroscopy, cyanide binds to the catalytic copper of Zn, Cu‐SOD thus preventing the interaction with the superoxide anion O_2_^‐^ [218]. This is in line with the observation that many copper‐dependent enzymes are indeed sensitive to cyanide, including CCOx (as discussed earlier).

Moreover, cyanide has been reported to inhibit zinc‐dependent enzymes such as alkaline phosphatase and carbonic anhydrase and this effect could be reversed by addition of Zn^2+^ ions, thus suggesting that cyanide inactivates the enzyme by removing of Zn^2+^ from the active site [219, 220]. The interaction of cyanide with gold has been reported to have therapeutic applications. Indeed, aurothiomalate, a gold complex used for the treatment of rheumatoid arthritis, has been suggested to be a prodrug of aurocyanide, the actual mediator of its anti‐rheumatic action, whose formation is promoted by MPO‐derived cyanide [206, 207]. A common cyanide antidote is cobalamin, in which a cobalt ion is complexed with a corrinoid ring. Cobalt(III), similarly to the iron heme, is able to bind both one and two molecules of cyanide, thus generating mono‐cyano and di‐cyano‐cobalamin [221]. The effectiveness of cobalamin as cyanide antidote is due to the fact it is able to bind cyanide with a greater affinity (K_A_ ∼ 10^12^ m ^−1^) [221] as compared to heme‐proteins, such as CCOx (K_A_ ∼ 10^7^ m ^−1^) [31].

Carbamylation is another, biologically relevant reaction catalyzed by cyanide in various cells and tissues. Carbamylation is a nonenzymatic post‐translational modification caused by the irreversible reaction of isocyanic acid (HCNO) with protein free amino groups, such as α‐NH_2_ group of terminal residues or ɛ‐NH_2_ group of lysine residues, although the latter occurs with ∼ 100 times lower rate than the former [222]. This post‐translational modification is associated with structural and functional alterations of target proteins such as tubulin, in which case it leads to the inhibition of microtubule assembly. Moreover, carbamylation has been associated with enzyme loss of function, as shown for superoxide dismutase, aspartate aminotransferase and matrix metalloproteinase‐2. Importantly, protein carbamylation has been associated with molecular aging, as well as a variety of diseases including atherosclerosis, chronic kidney disease, and rheumatoid arthritis [222, 223]. The biochemical pathways leading to formation of isocyanic acid are typically (a) spontaneous dissociation of urea into ammonia and cyanate [224] or (b) catalytic conversion of cyanide (or thiocyanate) to cyanate by MPO [225, 226]. As discussed above, this enzyme is abundantly expressed in inflammatory cells, such as neutrophils and monocytes, and its role in isocyanic acid formation has been associated with lipoprotein carbamylation and atherosclerotic plaques formation [227].

Cyanide is a typical ‘anthioanion’, a term referring to anions with a such high affinity for sulfur atoms that they form a stable thioanion when they react with elemental sulfur [228]. Indeed, cyanide is a strong nucleophile which readily reacts with sulfur–sulfur atoms through nucleophilic displacement, thus producing thiocyanate [229]. Protein disulfides are also susceptible to nucleophilic displacement in the presence of cyanide, thus generating a nonenzymatical post‐translational modification on protein cysteine residues called S‐cyanylation [230]. Although for a long time the occurrence of this protein post‐translational modification has been shown only *in vitro* using artificial conditions [231, 232], more recently it has been demonstrated that cyanide reacts with protein disulfide of human serum albumin and immunoglobulin G in a more physiologically relevant context [233].

As already mentioned in a previous section of our article, cyanide has been reported to target NMDA receptors by influencing the redox state of NR1/NR2A and NR1/NR2B channels, thus resulting in a modulation of their activity, yet with opposite effects. Indeed, while cyanide increased frequency and opening time of NR1/NR2A channels, it decreased opening frequency of NR1/NR2B channels. The latter modification is believed to be due to cleavage of the redox site ^744^Cys‐SS‐Cys^798^, leading to the formation of a thiocyanate adduct [65]. It is now well accepted that S‐cyanylation occurs naturally in plants, and it is believed to play a role in the modulation of crucial metabolic pathways, such as energy metabolism (glycolysis and TCA cycle) and S‐adenosyl methionine cycle. As shown in *Arabidopsis thaliana* roots extracts by employing two independent methodological approaches, such as liquid chromatography‐tandem mass spectrometry and 2‐iminothiazolidine chemical method, were identified 163 proteins susceptible to S‐cyanylation [4]. Among these, glyceraldehyde‐3‐phosphate dehydrogenase and isocitrate dehydrogenase have a high percent homology (∼ 70%) with human orthologs. Hence, it is conceivable that S‐cyanylation occurs in mammalian cells and tissues; This possibility remains to be explored in the future.

S‐glutathionylation is another post‐translational modification of protein cysteine residues by addition of glutathione. This modification has been shown to occur in several enzymes involved in energy metabolism, including α‐ketoglutarate dehydrogenase, isocitrate dehydrogenase, mitochondrial complexes I, II, and IV [27, 28]. S‐glutathionylation is often linked to oxidative stress (although it was identified in unstressed cells as well) by preventing irreversible oxidation of protein thiols along with modulating enzyme activity [27, 234]. Recently, we have reported that the S‐glutathionylated form of CCOx is less active as compared to its native form [29]. Interestingly, the de‐glutathionylation of CCOx could be obtained in the presence of nanomolar concentrations of cyanide, as shown both in HepG2 cell line and isolated enzyme from bovine heart [29]. The cyanide‐mediated de‐glutathionylation was responsible for the increase of CCOx activity, thus resulting in stimulation of cellular bioenergetics [29]. Mechanistically, this modification is a nucleophilic displacement, in which the disulfide cysteine residue covalently bounded to glutathione (Cys‐SSG) reacts with cyanide, thus promoting the restoration of the free cysteine residue and releasing glutathione as CN‐glutathione (CN‐SG) [29].

**Fig. 4:**
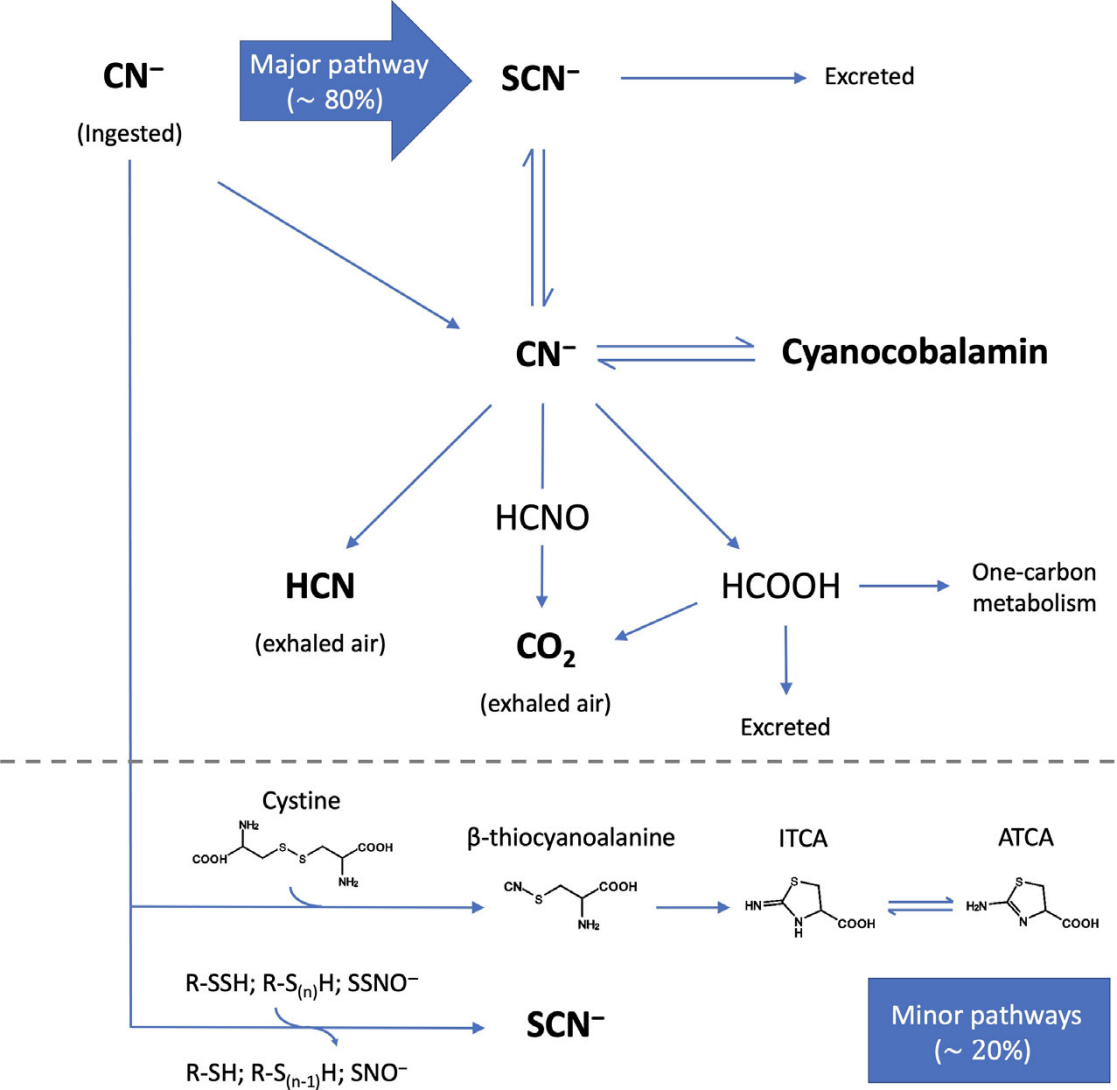
Reactions of cyanide in mammalian cells. CN^‐^ is a strong‐field ligand as well as a strong nucleophile. As such, it is involved in many reactions in mammalian cells. Metal binding*:* Cyanide is reactive toward transition metals, including iron, zinc, copper, and cobalt, thus binding many metalloproteins (often with an inhibitory effect) and the corrinoid ring of cobalamin. Prodrug activation: The prodrug aurothiomalate, used for the treatment of rheumatoid arthritis, is activated by cyanide and is believed that a CN‐gold complex is the actual mediator of its beneficial effect. Cyanohydrin formation: Due to its marked nucleophilicity, cyanide is particularly reactive toward electrophiles, such as carbonyl groups of aldehydes and ketones, thus forming cyanohydrin adducts. *PLP‐CN* complex formation: The reaction of cyanide with the aldehydic moiety of PLP has been shown to interfere PLP‐dependent enzymes. *NAD^+^‐CN* complex formation: The reaction of cyanide with the C‐4 of NAD^+^ has been shown to interfere with the activity of some dehydrogenases. Cyanylation: The reaction of cyanide with protein disulfide bridge leads to cyanylation, an emerging post‐translational modification suggested to be involved in the regulation of pivotal cellular pathways (*observation made in *Arabidopsis thaliana*). Carbamylation: HCNO has been shown to be involved in cellular aging by carbamylating target proteins, a post‐translational modification leading protein misfunctioning. De‐glutathionylation: Cyanide reacts with the cysteine‐glutathione disulfide of glutathionylated protein, thus displacing glutathione and restoring the protein thiol.

The principal biochemical pathways that are relevant for the reactivity of cyanide in mammalian cells are shown in Fig. 4. Additional, relatively poorly characterized reactions of cyanide in mammalian cells include its ability to react with potential additional reaction(s) with various protein cysteines, for instance in various membrane receptors [65, 67]. The strong nucleophilic character of cyanide makes it reactive toward electrophiles, such as carbonyl groups, thus generating cyanohydrin adducts by a nucleophilic addition. For instance, this has been reported for pyruvate and α‐ketoglutarate [130, 235], whose cyanohydrin conjugate may be used as a potential marker for cyanide exposure. A similar reaction has been reported for cyanide and pyridoxal 5’‐phosphate (PLP), as shown by UV‐Vis spectroscopy methods. Indeed, the presence of cyanide, a disappearance of a band at 388 nm was seen, characteristic of the aldehyde group in position 4 [236]. Working in homogenates of rat brain tissue, Cassel and colleagues observed a concentration‐dependent inhibition of the PLP‐dependent enzymes glutamic acid decarboxylase (GAD) and γ‐aminobutyric acid transaminase (GABA‐T). The enzymatic inactivation was due to the formation of a PLP‐CN complex, thus recalling the mechanism of action of classical PLP‐dependent enzyme inhibitors, such as amino‐oxy compounds [112]. However, the high IC_50_ value (280 µm for GAD) raises some doubt about the physiological relevance of this reaction.

Moreover, the possible interference of cyanide with substrates with carbonyl groups (such as α‐ketoglutarate and succinic semialdehyde, both substrates of GABA‐T) should be considered [112], thus making cyanide a ‘dirty’ inhibitor. This notwithstanding the binding of cyanide to PLP, at least in the free form, seems to occur in biological systems as shown by the ability of PLP to act as antidote for cyanide poisoning [237].

Direct reaction on the carbon C‐4 of the nicotinamide ring of NAD^+^ has also been reported, with different possible biological consequences. For instance, nicotinamide‐CN complex formation has been associated with stimulation of ADP‐ribosylation, as shown working on the mitochondrial fraction of rat liver [238]. This post‐translational modification is enzymatically regulated by ADP‐ribosyltransferases, which catalyze the cleavage of NAD^+^ and promotes the transfer of ADP‐ribose to target proteins. The resulting nicotinamide has been shown to be a potent inhibitor of ADP‐ribosyltransferases, and the complex nicotinamide‐CN may prevent the enzymatic inhibition [238]. Moreover, although a clear proof of the mechanism of action behind the cyanide‐induced increase of ADP‐ribosylation still lacks, the fact that cyanide reacts with the nicotinamide ring at pH ∼ 8 (which is close to the pH value of the mitochondrial matrix) or higher, may explain why cyanide did not produce any effect on cytosolic ADP‐ribosylation [238, 239]. The formation of NAD^+^‐CN complex has been suggested to be involved in the inhibition of NADH‐dependent enzymes such as alcohol dehydrogenase and formate dehydrogenase [240, 241]

Finally, higher concentrations of cyanide can produce significant shifts in the intracellular redox state, which, in turn, can affect a variety of cellular reactions, exemplified, for instance in shifts in the cellular NAD^+^/NADH balance [242], as well as various reactions with constituents of the biologically reactive sulfur pool (see below). The potential biological relevance of these reactions remains to be further investigated.

## Elimination of cyanide in mammalian cells and organisms

In nature, cyanide catabolism occurs mainly through five main pathways, which can be summarized as (a) hydrolytic pathway, (b) oxidative pathway, (c) reductive pathway, (d) syntheses pathway, and (e) substitution/transfer pathway as reviewed in [243]. The pathways from a to e are mainly spread in fungi and bacteria, while the substitution/transfer pathway is the only found in mammalians. This catabolic route involves three sulfur metabolizing enzymes, thiosulfate:cyanide sulfurtransferase (TST; also known as rhodanese), 3‐mercaptopyruvate sulfurtransferase (3‐MST), and sulfide:quinone oxidoreductase (SQR).

TST [EC2.8.1.1] is the main enzyme involved in cyanide detoxification via a reaction which was firstly described in 1933 by Lang *et al*., who observed in animal tissues the catalytic bioconversion of cyanide and thiosulfate into thiocyanate and sulfite [244]. The enzyme was then referred as rhodanese, from *rhodanid*, the German name of thiocyanate [244]. Its official name is thiosulfate:cyanide sulfurtransferase even if the name ‘rhodanese’ is still commonly used. The reaction catalyzed by TST takes place through a ping‐pong mechanism in which thiosulfate (S_2_O_3_^2−^) transfers a sulfur atom to a catalytic cysteine, to form a cysteine‐persulfide (Cys‐SSH) with the concomitant release of sulfite (SO_3_^−^). The reaction is completed when cyanide reacts with the Cys‐SSH, thus producing thiocyanate and regenerating the cysteine in the active site [245]. This reaction offers a rational behind the use of thiosulfate as antidote for cyanide poisoning. TST displays a catalytical efficiency in catabolizing cyanide in the order of 10^3^ m ^−1^ s^−1^, with cyanide showing K_M_ values of ∼ 30 mm, although polymorphic variants found in humans show a significant difference in sulfur transfer kinetics [246]. More recently, the role of TST in cyanide detoxification has been re‐evaluated because of its predominantly mitochondrial localization and relevant expression levels restricted to liver and kidney, thus leaving other tissue and organs more exposed to cyanide toxicity [247, 248, 249].

3‐MST [EC2.8.1.2], similarly to TST, is a sulfurtransferase and catalyzes the transfer of a sulfur atom to an acceptor. 3‐MST has been widely studied for its role in the biosynthesis of H_2_S, a small signaling molecule part of the family of gasotransmitters, along with CO and NO [250, 251]. Similarly to TST, 3‐MST catalyzes a reaction with double displacement mechanism in which 3‐mercaptopyruvate binds a cysteine in the active site, thus forming Cys‐SSH and eliminating pyruvate. The catalytic cysteine is then restored in the presence of a nucleophile which acts as a sulfur acceptor. Many compounds have been shown to be able, with different efficiency, to work as cosubstrates of MST, such as cyanide (K_M_ value of ∼ 6 mm) and in which case the catalytic efficiency is ∼ 400 m ^−1^ s^−1^ [252]. Although it has a catalytical efficiency one order of magnitude lower than TST, 3‐MST may play relevant role in cyanide detoxification because of its wider distribution in organs and tissues and its cytosolic localization (rather than merely mitochondrial) [253]. Consistently, are now under evaluation in animal model possible alternative antidotes than thiosulfate, aiming to exploit the 3‐MST catalyzed metabolic route. One promising approach seems to be the employment of prodrugs of 3‐mercaptopyruvate, because 3‐mercaptopyruvate itself appears to be too chemically unstable for such use [254].

SQR [EC1.8.5.8] is a flavoenzyme associated with the inner mitochondrial membrane and it catalyzes the first step of the detoxification pathway of H_2_S. Following its first identification in the lugworm *Arenicola marina* [255], the role of this enzyme in human physiology has been re‐evaluated with the discovery that it displays a sulfide oxidizing activity coupled with electron transfer along the electron transport chain, thus stimulating cellular bioenergetics [256, 257]. The reaction mechanism consists in the oxidation of H_2_S to sulfane sulfur, a reaction which involves two cysteine residues in the active site [258]. Similar to TST and 3‐MST, this reaction occurs with a ping‐pong mechanism in which the restoration of the free cysteine in the active site occurs in the presence of a nucleophile [259]. The catalytical efficiency of SQR in the presence of cyanide is in the order of 10^7^ m ^−1^ s^−1^, thus making of cyanide the most efficient sulfur acceptor for the SQR‐catalyzed reaction [260]. This notwithstanding, glutathione has been suggested to be the most physiologically relevant sulfide acceptor, despite the lower efficiency (10^4^ m ^−1^ s^−1^), because of the larger abundance in the cellular milieu (in the mm range) [261].

So far, TST has been viewed as the main cyanide detoxifying enzyme, and however, this role is most probably shared with 3‐MST. Although SQR has a catalytical efficiency in detoxifying cyanide 4 orders of magnitude higher than TST, its physiological role in cyanide catabolism has been considered negligible because of the low cyanide concentrations as compared to other sulfide acceptors (such as glutathione). However, the observation that cyanide can be catalytically biosynthesized within the mitochondrion [204] may reopen to a possible involvement of SQR‐mediated detoxification.

Approximatively 80% of cyanide metabolism is catalyzed by the three sulfur transferases, thus representing the main route of cyanide elimination (which is excreted by the kidneys as thiocyanate) [262]. However, because of cyanide’s reactivity, its metabolism is a complex process, which also involves a plethora of side reactions. As shown in an *in vivo* study, in which the radioactive ^14^C‐labeled sodium cyanide was used to track the metabolic fate of the carbon atom of cyanide in adult rats, it was concluded that (a) cobalamin is responsible in large part for the hepatic detoxification after acute exposure, as suggested by the high amount of cyanocobalamin detected in the urine the first day after the injection, and the subsequent rapid fall observed in the following days; (b) although the catabolic route leading to formation of thiocyanate overall represents the large part of cyanide catabolism, only two‐thirds of thiocyanate were found in the urines, thus suggesting that the remaining one‐third of the ^14^C of thiocyanate underwent further reactions before being excreted; (c) 1.7% of cyanide is eliminated by exhalation, of which the 90% in the form of CO_2_ and 10% in the form of free cyanide; (d) exists a metabolic equilibrium between cyanide and thiocyanate, as shown in rats by detecting an increase in the levels of exhaled hydrogen cyanide after treatment with radioactive thiocyanate, thus suggesting that thiocyanate can actually be converted to cyanide; (e) cyanide is involved in the one‐carbon metabolism, as shown by ^14^C detection in methyl groups of choline, methionine, formate, and a general distribution in protein and lipids [263].

Cyanide metabolism takes place mainly in the liver, which is the organ with highest abundance in rhodanase and cobalamin. A small amount of cyanide, although non‐negligible, takes part in various side reactions. For instance, the oxidation of cyanide to CO_2_ has been shown to be a viable metabolic pathway for detoxification and possibly implies the formation of a cyanate (CNO^‐^) intermediate. As shown *in vivo* experiments, the oxidation of cyanide to cyanate is catalyzed by superoxide dismutase [264], but also MPO may play an additional role [225]. The subsequent metabolic conversion of cyanate into CO_2_ has been well characterized in bacteria and plants, and it is catalyzed by cyanase. This enzyme is responsible for the conversion of cyanate, in the presence of bicarbonate as recycling substrate, into carbamate (H_2_NCOO^‐^), which decomposes spontaneously into CO_2_ and NH_3_ [265]. Although cyanase has not been found in higher organisms, but only in bacteria [266], it is conceivable that a similar reaction may also occur in humans.

Thiocyanate, commonly viewed as a metabolite of cyanide, is 100‐fold less toxic than cyanide and is excreted in the urine in about 2.7 days. Biological degradation of thiocyanate to release free cyanide under oxidative conditions (chlorination and ultraviolet disinfection) has been studied, mainly in the field of environmental biology [267]. But the observation that thiocyanate releases free cyanide in an *in vivo* system is somewhat unexpected. To our knowledge, the sulfurtransferase enzymes responsible for the bioconversion of cyanide into thiocyanide are not able to catalyze the reverse reaction (thiocyanate to cyanide). This has been confirmed by Goldstein and co‐workers, who first attempted to clarify the biochemical pathway behind the equilibrium between cyanide and thiocyanate. In an experiment carried out in different *in vivo* models (rats, dogs and rabbits), 3 h after the injection of thiocyanate (250–600 mg KSCN/kg), animals were euthanized, and different tissues were homogenized and analyzed for cyanide. Among the tissues studied, namely, liver, kidney, spleen, hearth, lung, striated muscle, stomach, brain and blood, only the latter was positive to the cyanide detection assay. Further experiments carried out also in human blood demonstrated that erythrocytes are the actual site for thiocyanate conversion to cyanide. Although the authors of the study were not able to unequivocally identify the enzyme responsible for this reaction, they named this enzyme thiocyanate oxidase [199]. Independent studies confirmed that cyanide generation could be detected within 10 min after the exposure of human blood cells (but not plasma) to thiocyanate [268]. In a follow‐up study, systemic fractionation of hemolysate showed that cyanide formation was present in all the fractions containing hemoglobin, and it was directly proportional the concentration of the enzyme. It was then clear that the oxidation of thiocyanate to cyanide in the erythrocyte is catalyzed by the peroxidase activity of hemoglobin rather than a more specific enzyme. The reaction occurred in the presence of hydrogen peroxide (H_2_O_2_) and led to the oxidation of thiocyanate to OSCN^‐^, O_2_SCN^‐^, and O_3_SCN^‐^ and eventually releasing sulfate and cyanide. This reaction has been shown to be catalyzed by the enzyme in the form of methemoglobin, whose peroxidase activity was relatively high in crude hemolysate (working with excess of H_2_O_2_), but low in erythrocyte, possibly because of the low concentration of H_2_O_2_ in the cellular milieu [198].

The finding of the potential involvement of cyanide in the one‐carbon metabolism suggests that this molecule is, to some extent, a metabolically active compound. This opens the possibility to a link between cyanide metabolism, the methionine cycle and folate cycle which in turn provide one‐carbon units (in the form of methyl groups) for the synthesis of lipids, proteins and DNA. The metabolic link between cyanide and one‐carbon metabolism could be formate, a nontetrahydrofolate‐linked intermediate of the one‐carbon metabolism [263, 269]. Although the involvement of cyanide in the one‐carbon metabolism seems to be generally accepted as witnessed by a number of reviews mentioning this pathway [266, 270, 271, 272], a fundamental—and so far unanswered—question is how cyanide is converted to formate in humans. So far, the metabolic sources of formate in humans are mainly serine and glucose, but also choline, methanol, and formaldehyde [273]. Formate is known to be a metabolic mediator mammals and gut microbiota. Indeed, some bacterial species populating the intestinal flora are able to produce formate through anaerobic fermentation [273]. Interestingly, hydrolytic conversion of cyanide into formate is catalyzed by cyanidase (cyanide dehydratase), an enzyme found in many bacteria, fungi, yeasts and plants [271, 272].

As already discussed, cyanide has a marked reactivity for sulfur‐containing molecules, particularly for disulfides (R‐SS‐R) and polysulfides (R‐S_n_H). A similar reaction has been described for cystine, in which case the cyanide‐mediated cleavage of the disulfide bond leads to formation of β‐thiocyanoalanine intermediate, which undergoes an intramolecular nucleophilic attack with consequent formation of 2‐amino‐2‐thiazoline‐4‐carboxylic acid, a compound which is currently used as biomarker of cyanide exposure and employed for forensic purposes [179, 180].

**Fig. 5:**
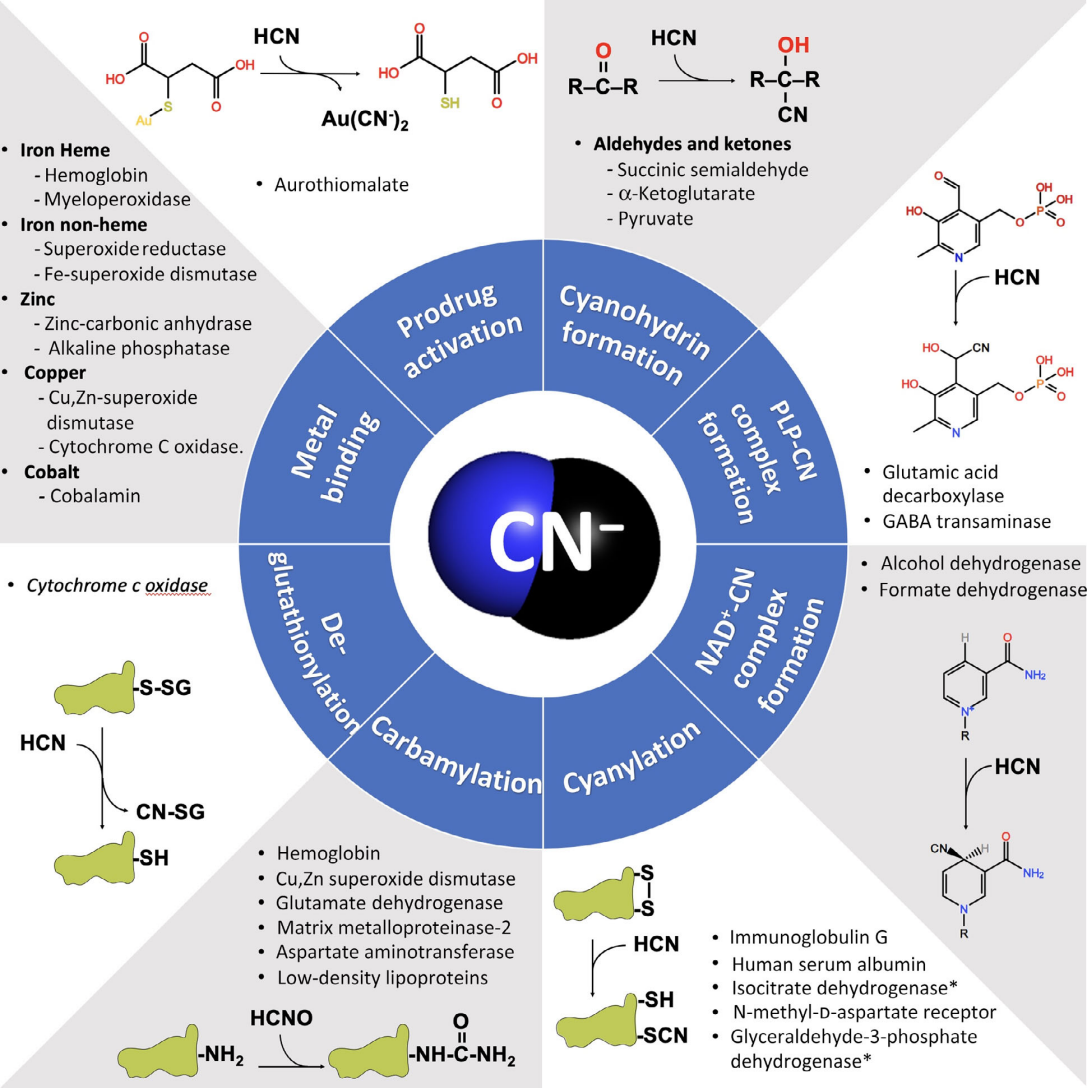
Elimination pathways of cyanide. CN^‐^ is manly catabolized into thiocyanate (SCN^‐^) by the sulfurtransferase enzymes and excreted as such in the urine. SCN^‐^ exists in a metabolic equilibrium with the cyanide pool. The possible metabolic fates of the cyanide pool include i) interaction with cobalamin, thus forming cyanocobalamin; ii) conversion to formate, which, in turn, can be exhaled as CO_2_, excreted in the urine, or take part to the one‐carbon metabolism; iii) conversion to HCNO and consequent exhalation as CO_2_; iv) exhalation of free CN^‐^. Minor pathways involve v) a reaction with cystine and consequent generation of 2‐iminothiazolidine‐4‐carboxylic acid (ITCA), in tautomeric equilibrium with ATCA, and vi) various additional reactions with reactive sulfur species such as persulfides, polysulfides, and nitrosopersulfides.

The principal biochemical pathways that are relevant for the metabolism and elimination of cyanide in mammalian cells are shown in Fig. 5.

## Cyanide as a physiological regulator in mammalian cells

In the previous sections, several pathways and mechanisms have been described that result in cyanide production by mammalian cells. The best characterized of these mechanisms being the neutrophil‐derived cyanide generation (triggered by neutrophil activation and oxidative burst), and cyanide generation in neurons (a process that generates cyanide basally, in addition, cyanide production can be further enhanced by opioid receptor stimulation). The underlying enzymatic reactions have been outlined in the previous section. However, the question, as to whether cyanide, at endogenous levels, may serve biological regulatory roles has not been explored extensively; the vast majority of the studies exposed cells to cyanide in high (hundreds of micromolar to low millimolar) concentrations and have focused on deleterious effects, such as impairment of bioenergetics and cell death (apoptosis at lower concentrations and necrosis at higher concentrations). Although in the intermediate concentration range, cyanide was found to activate various cellular signaling pathways (e.g., calcium mobilization), these processes were also predominantly investigated in the context of activation of deleterious pathways (e.g., caspases and endonucleases, inducing DNA fragmentation and apoptosis). Nontoxic cyanide‐associated responses that may be best characterized as potentially physiologically relevant were the enhancement of NMDA receptor activation (occurring at 50–100 µm cyanide) [64, 65, 66, 67] (given the fact that NMDA receptors play multiple physiological roles) and perhaps a selective stimulation of neurotransmitter release at relatively noncytotoxic concentrations (100 µm) of cyanide from neurons [274]; this action may also be relevant in physiological contexts.

However, three independent lines of more recent studies have extended the investigations of the cellular effects of cyanide into a lower concentration range (nanomolar to low micromolar). These studies support the view that low concentrations of cyanide can exert cytoprotective and beneficial effects in mammalian cells and tissues. (a) Isom’s group has investigated the effect of cyanide in PC12 cells *in vitro* and demonstrated that cyanide, in the concentration range of 1–10 µm, causes a rapid‐onset, approximately 50% elevation of intracellular IP_3_ levels. The proposed mechanism involves an early step of extracellular calcium influx into the cells (via a mechanism which remains to be characterized). This calcium signal, in turn, activates phospholipase C, which, in turn, induces IP_3_ release [68]. IP_3_, in turn, mobilizes intracellular calcium via its well‐known mechanism of action (binding to its receptor on the endoplasmic reticulum and opening calcium channels in its membrane) [275, 276]. IP_3_ is an essential regulator of essential signaling processes, influencing essential cellular functions in health and disease [275, 276]. Thus, via regulation of IP_3_ at low (likely physiological) concentrations, cyanide can potentially regulate a variety of pathways (e.g., a variety of calcium‐dependent intracellular enzymes and signal transduction pathways, the Akt‐signaling cascade, G‐protein‐coupled receptor kinase‐interacting proteins 1 and 2, mTOR‐controlled autophagy pathway, the Bcl‐2‐family members) and associated processes (ER stress, survival, cell death, gene transcription etc.)

(b) In an independent line of studies, Correira and colleagues exposed RBE4 rat brain endothelial cells and NT2 neuron‐like cells to cyanide in the concentration range of 10 nm–1 µm and observed an increase (rather than the usual decrease noted with cytotoxic levels of cyanide) in oxygen consumption of the mitochondria [277]. Already at these low concentrations of cyanide, a slight increase in mitochondrial ROS generation was noted, as well as a detectable rearrangement of mitochondrial ultrastructure [277]. Importantly, exposure of the cells to cyanide (at 100 nm and 1 µm concentrations) induced a significant increase of HIF‐1α expression in endothelial cells, as well as an induction of eNOS, VEGF and erythropoietin expression, and increased the ratio of mitochondrial/cytosolic BAX, suggesting that low concentrations of cyanide can affect gene expression and can induce a preconditioning‐type response, which protected the cells from a subsequent cytotoxic insult [277]. These data indicate that cyanide, at nanomolar and low micromolar concentrations, exerts regulatory and cytoprotective, rather than cytotoxic roles in mammalian cells.

(c) Our group has recently investigated the effect of low (nanomolar to micromolar) concentrations of cyanide in HepG2 cells (a human hepatoma line) and observed that cyanide at low (0.1 nm–1 µm) concentrations stimulates mitochondrial electron transport and oxygen consumption, while higher (10 µm and above) concentrations produce the well‐established inhibitory effects. Low concentrations of cyanide rapidly (within minutes) increased cellular ATP levels, as confirmed by two independent methods (Extracellular Flux Analysis and a ratiometric intracellular ATP:ADP fluorescent biosensor); the effects of cyanide were attributed to a direct activation of mitochondrial Complex IV (while the activity of Complexes I, II, and III were largely unaffected by cyanide) [57]. The mode of Complex IV activation was attributed to a cyanide‐mediated de‐glutathionylation of cytochrome C oxidase (via a biochemical mechanism discussed in a previous section) [29]. Low concentrations of cyanide also induced a detectable stimulation of cell proliferation in HepG2 cells as well as in a variety of other human cell lines [29]. The expression of various cyanide‐producing and cyanide‐metabolizing enzymes was also confirmed in HepG2 cells; interestingly, one of the cyanide‐producing enzymes, myeloperoxidase, underwent a biphasic expression change with cyanide: low concentrations induced an upregulation, while higher concentrations produced a downregulation [29].

Taken together, the above studies indicate that low concentrations of cyanide (in the concentration range that is relevant in physiological context) exert significant effects in mammalian cells; these effects include the stimulation of bioenergetics, the stimulation of cell proliferation, and confer a cytoprotective phenotype. Moreover, low, noncytotoxic concentrations of cyanide can significantly affect the expression of multiple gene products, raising the possibility that cyanide (via redox mechanisms, or other pathways) may act as a global regulator of gene expression. The induction of HIF‐1α by high (cytotoxic) concentrations of cyanide has been previously observed [278], but the ability of low (nontoxic) concentrations of cyanide to regulate the expression of this factor may be particularly significant in the context of physiological cell responses.

The studies of Hosseini and colleagues [279] may also indirectly support the concept that endogenous cyanide, produced by MPO, may exert stimulatory effects on cellular bioenergetics: these studies, conducted in a tumor cell line, demonstrated that MPO silencing decreases (rather than increases) the cellular bioenergetic profile. One potential interpretation of these findings may be that endogenous, MPO‐derived cyanide exerts a basal stimulatory effect on mitochondrial function and cellular bioenergetics.

**Fig. 6:**
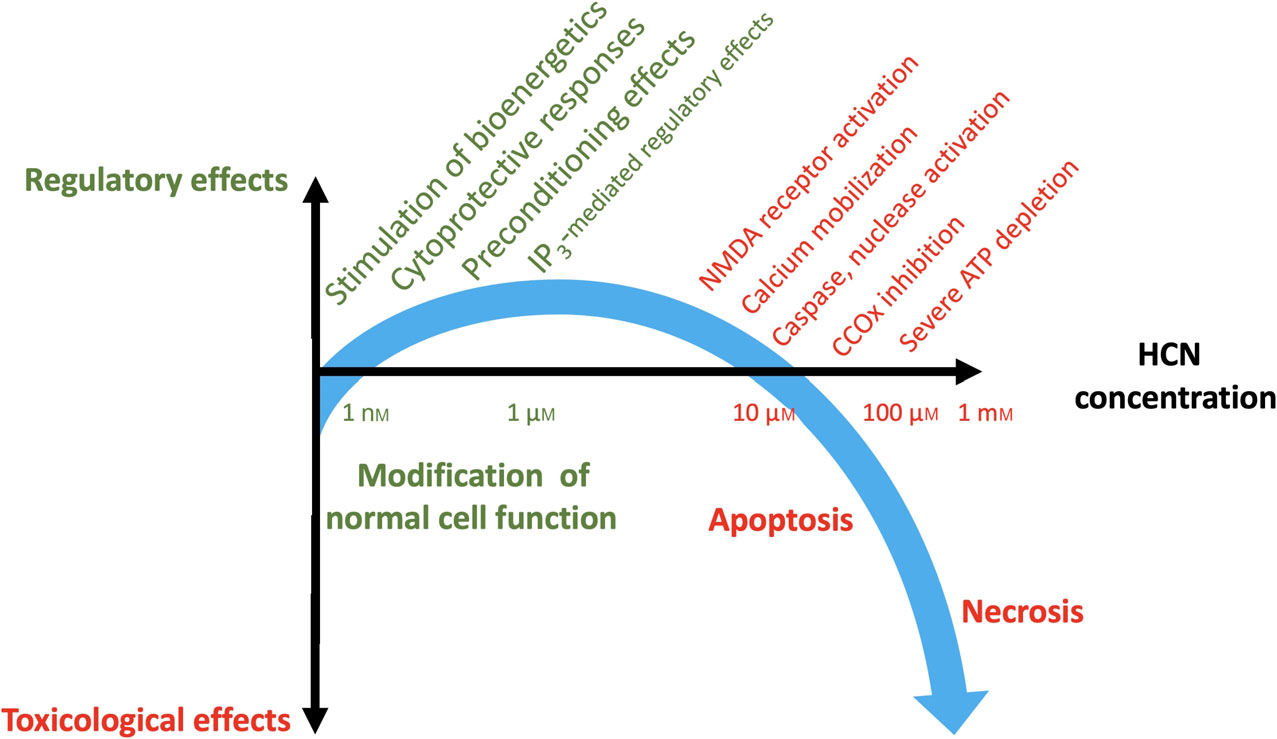
Role of low and high cyanide concentration in the regulation of mammalian cell function in health and disease. At low (nanomolar to low micromolar) concentrations, cyanide exerts beneficial and regulatory roles, for example, stimulation of cellular bioenergetics, induction of a cytoprotective phenotype. At medium concentrations, the effect of cyanide includes the regulation of NMDA receptors and the modulation of intracellular calcium handling, which may either exert physiological or pathophysiological roles, depending on the circumstance. At high (high micromolar to millimolar) concentrations, cyanide induces calcium overload and mitochondrial dysfunction, culminating in apoptotic, or necrotic cell death.

The spectrum of cyanide’s actions in the physiological and pathophysiological contexts, and the associated concentration ranges, are shown in Fig. 6.

## Cyanide as a potential novel mammalian gasotransmitter

There is a set of generally accepted criteria that applies to *mammalian gasotransmitters*. [280, 281, 282, 283, 284]. According to these criteria, (a) gasotransmitters are small molecules of gas (i.e., they exist in gaseous form or are dissolved in the extracellular or intracellular fluid). They have a small molecular weight, but their derivatives can have different molecular weights and may not necessarily be in gas state. Regardless, these derivatives are still part of the gasotransmitter family. For example, NO qualifies as a gasotransmitter, but also its nongaseous derivatives such as nitrite, nitrate, HNO, or peroxynitrite. Likewise, H_2_S is a gaseous molecule, but also its nongaseous derivatives (e.g., polysulfides or thiosulfate) are members of the family. As discussed earlier in the current review, cyanide (HCN) clearly fulfills this criterion, since it is largely in the gaseous state in physiological fluids. (b) Gasotransmitters readily cross cellular membranes. Thus, their movements are not restricted by transporters or membrane receptors, and their cellular actions can involve multiple receptors and targets. As discussed earlier in the current review, cyanide also fulfills this criterion, since it is readily diffusible and has multiple molecular targets. (c) Gasotransmitters are the products of mammalian cells and tissues; they are generated by mammalian enzymes, utilizing endogenous substrates. As discussed earlier at least one enzyme, myeloperoxidase, generates cyanide from endogenous substrates and thus fulfills this criterion. There are also other mammalian enzymes and processes that generate cyanide, for instance from alimentary sources. It is conceivable that future research will identify additional mammalian cyanide‐producing enzymes. (d) Importantly, gasotransmitters are expected to be biologically generated in a *regulated* manner. As discussed earlier in the current review, cyanide already meets this criterion, since it is produced in various cell types in regulated manner (e.g., in neurons, opioid receptors, and muscarinic receptors regulate its generation); moreover, it is readily diffusible and has multiple molecular targets. However, more work remains to be done in this area to characterize the pathways and mechanisms of regulated cyanide production. (e) Finally, gasotransmitters are expected to fulfill signaling (i.e., biological and physiological) functions (as opposed to toxicological effects) in mammalian cells, at physiologically relevant concentrations; it is also expected that the function of a gasotransmitter can be mimicked by their exogenously applied counterparts. For a long time, cyanide was not considered to fulfill this criterion, since its effects were primarily considered in the toxicological context only. However, as reviewed in the previous sections, there are several independent lines of more recent data showing that cyanide in the nanomolar to single digit micromolar concentrations exerts biological effects that are of regulatory nature (e.g., IP_3_ release, stimulation of intracellular calcium mobilization, cytoprotection, regulation of gene expression and stimulation of mitochondrial electron transport and elevation of intracellular ATP levels). Admittedly, the available body of evidence related to the effects of cyanide in this low (nanomolar‐to‐low micromolar) concentration range is currently relatively small; we believe one of the reasons is that cyanide was not considered as a physiological regulator and its effects at this low concentration range were usually not considered or evaluated.

The current evidence discussed above, as well as another recent commentary [285] suggests that cyanide may be a viable candidate as a novel endogenous gasotransmitter. Nevertheless, much additional work remains to be done in this area (including the better characterization of endogenous cyanide concentrations in various cells and tissues). Also, substantial work remains to be done focusing on the effect of scavenging endogenous cyanide production or inhibiting or silencing the various endogenous cyanide‐producing or cyanide‐metabolizing enzymes, to unveil the (likely) dynamic regulation of cyanide by the combination of regulated biosynthesis and multiple pathways of metabolism and elimination.

Other common features of gasotransmitters (including the potential ‘recent addition’, cyanide) are summarized in Table 2. (Ammonia, and methane, which can also be considered as gaseous mediators, with some caveats, that is ammonia does not appear to have a clear physiological role in mammalian cells and methane does not appear to be generated by mammalian cells from—are discussed in a separate article [284]). These features include the fact that their production occurs across broad representatives of the animal and plant kingdom (including bacteria), relatively short half‐life. Typically, these features also include biphasic or bell‐shaped effects, whereby high concentrations of these molecules are toxic (while low/endogenously produced concentrations are regulatory). It is the case for NO, CO, H_2_S, and now cyanide, that these toxicological effects were recognized first, and the biological roles and endogenous production was only discovered later. All of the known gasotransmitters have important roles in the regulation of mitochondrial and cellular bioenergetic functions. The gasotransmitter‐mediated regulation of mitochondrial function makes a lot of evolutionary sense, given the fact that bacteria already have multiple pathways to generate these mediators, and mitochondria are, from an evolutionary standpoint, modified bacteria.

**Table 2:** Comparison of the biological production and cellular action of the gasotransmitters nitric oxide (NO), carbon monoxide (CO), hydrogen sulfide (H_2_S), and a potential additional gasotransmitter, hydrogen cyanide (HCN)

	NO	CO	H_2_S	HCN
Initially characterized as a toxic gas, an environmental hazard?	Yes, it was known to be produced in exhaust fumes from internal combustion engines. A constituent of cigarette smoke.	Yes, it has been long known as a toxic gas emitted from partial combustion of organic molecules; from internal combustion engines, volcanic eruptions, forest fires, household furnaces. A constituent of cigarette smoke.	Yes, it has been recognized as a toxic gas emanating from sewers, swamps and as a toxic byproduct of processes used in paper mills and other industrial processes. Also produced by the normal enteral bacterial flora.	Yes, it has been recognized as a toxic gas used as warfare agent and inhaled during housefires. It is also known as a toxic byproduct of various industrial processes. Also produced via the metabolism of certain alimentary plants.
Produced by plants?	Yes, via reduction of nitrite to NO via different nonenzymatic or enzymatic mechanisms.	Yes, via various heme oxygenase family members.	Yes, via cysteine desulfhydrases and O‐acetyl‐L‐serine (thiol) lyase	Yes, as a (by)product in ethylene and camalexin synthetic pathways and viaβ‐glucosidase from glucosidic cyanogens.
Produced by bacteria?	Yes, via nitrite reductases and NO synthases.	Yes, via various heme oxygenase family members.	Yes, by bacterial isoforms of CBS, CSE and 3‐MST and several other pathways.	Yes, via glycine decarboxylation by HCN synthase.
Subsequently discovered as a molecule produced by mammalian cells?	Yes, it is produced from L‐arginine by a class of enzymes: nitric oxide synthases (NOS1, NOS2, NOS3). It is also produced by a variety of nonenzymatic processes (e.g., from nitrite).	Yes, it is synthesized from heme, as a product of a class of enzymes: heme oxygenases.	Yes, it is synthesized from L‐cysteine, as a product of CBS and CSE and from 3‐mercaptopyruvate via 3‐MST. It can also be produced via nonenzymatic processes.	Yes, it is synthesized by various mammalian enzymes, for instance myeloperoxidase and likely by other peroxidases. It can also be produced via nonenzymatic processes.
Mammalian endogenous substrate(s)	L‐arginine	Heme	L‐cysteine, homocysteine, 3‐mercaptopyruvate	L‐glycine (and likely others)
Pharmacological inhibitors of its production	N‐substituted L‐arginine derivatives, for example, N^G^‐methyl‐L‐arginine (L‐NMMA), guanidine derivatives (e.g., aminoguanidine), many others.	Heme oxygenase inhibitors, for example, typically Zn‐protoporphyrin‐IX.	Aminooxyacetate, beta‐cyano‐L‐ alanine and DL‐propargylglycine for CBS and CSE and HMPSNE for 3‐MST.	Remain to be refined; one possible class of inhibitors may be myeloperoxidase inhibitors.
Diffusible, labile gas?	Yes; breakdown products include nitrite and nitrate. Half‐life: seconds. Elimination: via the urine as nitrite and nitrate; also via exhaled air.	Yes. Half‐life: hours. (Half‐life as CO‐Hb: minutes). Elimination: via exhaled air.	Yes, breakdown products include thiosulfate, sulfite and sulfate. Half‐life: seconds to minutes. Eliminated: via the urine and via exhaled air.	Yes, breakdown products include thiocyanate. Half‐life: minutes to hours. Eliminated: via the urine and via exhaled air.
Reacts with hemoglobin?	Yes; to yield nitrosyl‐hemoglobin and methemoglobin.	Yes, to yield carboxy‐hemoglobin.	Yes, to yield sulfhemoglobin.	Yes, to yield cyanmethemoglobin.
Free radical?	Yes.	No.	No.	No.
Its ‘receptor’.	Guanylate cyclase with high affinity, thiol groups, heme groups; K_Ca_ channels; various other targets.	Guanylate cyclase (with lower affinity than NO); K_Ca_ channels; various other targets.	K_ATP_ channels; phosphodiesterases, various other targets.	NMDA receptors; calcium channels, various other targets.
Does it affect cellular bioenergetics?	Yes, via cytochrome c oxidase and other mitochondrial enzymes and after conversion to peroxynitrite, via the nuclear enzyme poly(ADP‐ribose) polymerase.	Yes, via inhibition of cytochrome c oxidase and others.	Yes, via inhibition of cytochrome c oxidase and others. At high concentrations it can induce metabolic suppression in cells.	Yes, via cytochrome c oxidase (stimulation at very low concentration; inhibition at higher concentration). Its best documented biological and toxicological effect is a metabolic suppression of mammalian cells.
Vasodilatory effects?	Yes, via stimulation of the guanylate/cGMP system, K_ATP_ channel opening, and additional mechanisms.	Yes, via stimulation of the guanylate/cGMP system and additional mechanisms.	Yes, via K_ATP_ channel opening, stimulation of the cGMP system and additional mechanisms.	Yes, at rather high concentrations, most likely via energetic paralysis.
Anti‐inflammatory and cytoprotective effects?	Yes, at low concentrations. Higher concentrations are toxic via metabolic inhibition directly or via the formation of secondary products (e.g., peroxynitrite, and subsequent activation of DNA injury, mitochondrial dysfunction and other cell injury pathways).	Yes, at low concentrations, involving MAP kinases and other pathways. Higher concentrations are toxic via metabolic inhibition.	Yes, at low concentrations. Higher concentrations are toxic via induction of metabolic inhibition.	Yes, cytoprotective effects at very low concentrations. Higher concentrations are toxic.

One of the more contentious issues in gasotransmitter biology is the question of ‘absolute concentrations’ of these mediators in biological matrices. With respect to cyanide, these concentrations are probably in the low micromolar/high nanomolar range, but these values are dependent on the methods used. Similar to cyanide, the absolute concentrations of H_2_S are a matter of ongoing debate, and even the concentrations of CO and NO remain to be further defined. The difficulties in determining these values are partially methodological, partially related to the nature of these molecules. For instance, the reported values for NO and H_2_S depend on the ability of the method to include or exclude various reactive NO ‘pools’ or reactive sulfur ‘pools’ in the assay. Regardless of the nature of the method, the concentrations of ‘free’ NO, CO, H_2_S, and HCN are probably in the low µm or high nm range; clearly, in these concentrations these molecules are not deleterious to the viability of the cell and serve biological purposes.

## Multiple interactions exist between various gasotransmitters

In serving these regulatory roles, there are many interactions between the various gasotransmitters; in addition, they also often interact with various reactive oxygen species. Cyanide, for instance, appears to interact with the NO system in several ways: NO modulates the ability of cyanide to inhibit CCOx [39, 60]; moreover, low concentrations of cyanide upregulate NOS expression [277]. Moreover, cyanide appears to interact with the H_2_S system through a variety of actions. Thiocyanide can be nonenzymatically produced from the reaction of cyanide and elemental sulfur, polysulfide, or sulfide, or, via the catalysis of 3‐MST, it can be generated from cyanide and thiosulfate (a H_2_S metabolite).

Also, importantly, 3‐MST is not only a cyanide‐metabolizing enzyme, but also a H_2_S producing enzyme. 3‐MST is an oxidant‐sensitive enzyme; thus, oxidative inactivation (or downregulation) of 3‐MST would not only be expected to suppress cyanide detoxification (to increase its levels), but it would also be expected to inhibit H_2_S/persulfide biosynthesis. Of interest, persulfides are prone to directly react with nucleophiles such as cyanide, thus forming thiocyanate [258]. Because of their disulfide bond, also the hybrid S/N species nitrosopersulfide SSNO^‐^ can react with cyanide to give SNO^‐^ and SCN^‐^ [286, 287]. Thiocyanide, can, in turn, regenerate cyanide (for instance via a reaction with hydrogen peroxide). The cold cyanolysis (i.e., nucleophilic attack on a sulfur–sulfur bond by cyanide) [288] may also occur in biological systems with potentially diverse functional consequences. Recently, trisulfide intermediate formation has been observed during the catalytic turnover of SQR, a sufide‐catabolizing enzyme which can use cyanide as cosubstrate. Particularly, it has been shown that this enzyme harbors in its active site a cysteine trisulfide and, according to the model proposed by the authors, cyanide interacts with the catalytic trisulfide and removes one sulfur atom by a cyanolysis reaction [289].

The interconnection of the metabolic pathways of cyanide and H_2_S may involve also other H_2_S‐sythesizing enzymes than MST. For instance, cystathionine γ‐lyase (CSE), along with CBS, have been shown to be a source of hydropersulfides (RSSH), and their upregulation besides offering protection against oxidative damage [290] may boost cyanide detoxification *via* both a direct and indirect pathway. For instance, in an *in vitro* enzymatic coupled assay, it was shown that CSE greatly enhanced the rhodanese‐catalyzed bioconversion of cyanide into thiocyanate, by generating cysteine trisulfide (or thiocystine; Cys‐SSS‐Cys), which serves as a sulfur donor substrate for rhodanese [291]. Interestingly, thiocystine‐dependent rhodanese activity has been shown to be 7‐fold higher than that in the presence of thiosulfate [289]. Consistently, when injected intravenously, thiocystine protected adult rats from cyanide poisoning [290]. Moreover, as shown in a panel of cell lines, thiocystine proved a marked protection against electrophilic stress, possibly by increasing the bioavailability of pesrulfidated species such as Cys‐SSH and GSSH [291]. In turn, persulfides readily react with oxidants such as H_2_O_2_, resulting in the formation of the unstable intermediate alkyl perthiosulfenic acid (RSSOH), which, in the presence of thiol compounds, leads to regeneration of trisulfide species (R‐SSS‐R) [292]. Thiocystine has been also shown to directly interact with cyanide thus generating Cys‐SSH and Cys‐SCN [293, 294, 295]. Both CSE and CBS are PLP‐dependent enzymes and, although cyanide has been shown to directly react with PLP, thus leading to enzyme inhibition [118], there are no reports suggesting a such reaction in the case of CSE and CBS. The presence of a heme prosthetic group makes the latter enzyme a potential target for small molecule such gasotransmitters, as shown for NO and CO [296]. However, despite the similarities shared by these small ligands, CBS is not likely to be targeted by cyanide at physiological conditions, as suggested by the high K_d_ values (mm range) [297].

There may be complex interactions between NO, H_2_S and cyanide on protein cysteines via a combination of nitrosylation, sulfhydration and, possibly, cyanylation reactions; moreover, as discussed earlier, cyanide (after cyanate conversion) can also induce carbamylation of amino groups (ɛ‐amino groups of lysine residue side chains) in proteins. There may be also various interactions on protein glutathionylation; as discussed earlier, cyanide can remove glutathione groups from proteins, while the endogenous, NO‐derived nitrosothiol S‐nitrosoglutathione can promote protein glutathionylation [27, 28, 29, 234]. Clearly, the already complex interactions within the so‐called “reactive species interactome” [298] will be made even more complex by the addition of cyanide as another physiological and pathophysiological interactor.

## Future directions

The traditional areas of cyanide‐related research focus on three areas. (a) The first area is the mechanism of cyanide cytotoxicity and organ toxicity. This area has a clear relevance for environmental toxicology and other areas of biology connected to various forms of cyanide intoxication. As discussed in the previous sections, work in this area (utilizing direct administration of exogenous cyanide to cells, isolated organs or animals) has identified a significant number of interrelated mechanisms, although full integration of the various pathophysiological pathways and mechanisms remains to be completed. One of the questions that remains to be further clarified, is how much of cyanide cytotoxicity is downstream of mitochondrial inhibition, and how much is triggered by actions of cyanide on other cellular effectors. Much more work remains to be performed in the field related to the role of cyanide in the bacterial–mammalian cell interactions, for example, in the context of cystic fibrosis (where, for instance, direct studies attempting to neutralize bacterial cyanide generation) are missing. In other potential *Pseudomonas*‐associated diseases (e.g., urinary tract infection or systemic inflammation / sepsis), there are presently no direct studies, although the experimental tools (strains of cyanide‐production deficient *Pseudomonas*, cyanide scavengers, etc.) are readily available. (b) The second area of future investigations relates to the field of the generation of cyanide‐sensitive probes and improved methods to detect cyanide in biological fluids or in cells. Given its potential toxicity and environmental hazard, many techniques for cyanide detection have been developed, ranging from rough methods such as necked eye detection methods to more quantitative techniques like gas chromatography/mass spectrometry. This notwithstanding, few methods combine high levels of sensitivity, selectivity, and ease of application, as reviewed in [299]. Overall, as compared to colorimetric techniques, fluorescent‐based detection methods offer a higher sensitivity, with a detection limit which can reach the order of 10 nm. However, the selectivity studies for these probes are carried out only toward few compounds such as F^−^, Cl^−^, Br^−^, I^−^, just to mention a few, while the cellular milieu is a complex matter and interference with other strong nucleophiles not always can be predicted, for this reason colorimetric methods are still a good alternative. Since methods for the detection of cyanide in biological systems (e.g., cells in culture) are less developed, and a gold standard cyanide probe, which is selective, sensitive and cell permeable (i.e., suitable to detect endogenously produced cyanide in living cells) remains to be established. (c) The third traditional area of cyanide research relates to the improvement of pharmacological tools and clinically used drugs suitable for the therapy of cyanide intoxication. As discussed elsewhere [14, 15], there are many classes of drugs or potential drug development candidates for this purpose, but the efficacy of these agents needs to be improved and the therapeutic window of administration needs to be extended. In this respect, it would be important to take a renewed focus on potential fresh and new approaches that may be suitable to displace cyanide from CCOx to reactivate mitochondrial electron transport and aerobic ATP generation.

(d) For at least three decades now, significant interest has been generated related to the potential anticancer effect of various plant‐based cyanogenic glycosides, for instance, laetrile or amygdalin (contained in apricot kernels). However, comprehensive meta‐analysis reveals that the potential efficacy of laetrile or amygdalin in cancer patients is not currently supported by sound clinical data [300, 301, 302]. At the same time, there is a significant risk of serious adverse effects from cyanide poisoning after laetrile or amygdalin administration [300, 301, 302]. Although the above approach, as a therapeutic direction, does not appear to bear fruit, it is clear from multiple *in vitro* studies that cancer cells can be killed or metabolically inhibited by either cyanogenic glycosides and/or by authentic cyanide [303] (although the concentrations required for such effects do not appear to be different from the concentrations required for the same effect in normal cells). Nevertheless, the possibility remains that there may also be some potential future utility in exploiting the toxicological effects of cyanide in the context of tumor therapy, especially if future approaches could selectively target the cytotoxic action of cyanide to the tumor cells and tumor tissue; there are some preclinical studies that produced encouraging data in this direction [304, 305].

Regarding more novel, and nontraditional areas, (e) there should be a new interest to further investigate the biological effects of low (noncytotoxic) concentrations of cyanide in various biological systems. These studies could focus on cytoprotective effects, the regulation of mitochondrial function and global cellular metabolomics, and perhaps the ability of low‐dose cyanide to produce a safe and effective method for a preconditioning‐type cytoprotective effect. The pathways and effectors involved in the effects of low‐dose cyanide in various cell types (in health and disease) also remain to be established. Likewise, (f) the biochemical reactions involved in the effects of cyanide in biological (regulatory) contexts remains to be further studied. For instance, does cyanide induce cyanylation in mammalian cells and tissues? (All of the required biochemical effectors for this post‐translational modification appear to exist in mammalian cells.) (g) Additionally, the question whether cyanide can induce de‐glutathionylation of targets other than CCOx should be investigated. Glutathionylation is an emerging field of post‐translational protein modification, with significant roles in cell signaling and cell metabolism; it is conceivable that cyanide interacts with this system in multiple ways. (h) Moreover, the pathways and enzymes involved in endogenous (mammalian) cyanide generation should be further investigated. How are the known cyanide‐producing enzymes (e.g., myeloperoxidase) regulated in biological contexts? Are there conditions where the known cyanide‐degrading enzymes may work in ‘reverse’ and may contribute to the generation of cyanide in mammalian cells? Are there additional cyanide‐producing mammalian enzymes? What are the exact pathways and enzymatic reactions that contribute to receptor‐mediated or receptor‐regulated endogenous cyanide generation in neurons? How do the above mechanisms become affected in various diseases, or during physiological aging? (i) Finally, how does endogenously produced cyanide interact with the other gasotransmitter systems on already known targets (CCOx, globins, etc.) or on other common cellular targets in health and disease? Further work in these areas is expected to clarify this question and may open a new direction in gasotransmitter biology.

## Conflict of interest

The authors declare no conflict of interest.

## Author contributions

CS conceived and CS and KZ wrote the manuscript.
